# The Human *SCN9A*^*R*185*H*^ Point Mutation Induces Pain Hypersensitivity and Spontaneous Pain in Mice

**DOI:** 10.3389/fnmol.2022.913990

**Published:** 2022-06-13

**Authors:** Yaping Xue, Mélanie Kremer, Maria del Mar Muniz Moreno, Celeste Chidiac, Romain Lorentz, Marie-Christine Birling, Michel Barrot, Yann Herault, Claire Gaveriaux-Ruff

**Affiliations:** ^1^Centre National de la Recherche Scientifique (CNRS), Institut de la Santé et de la Recherche Médicale (INSERM), Institut de Génétique et de Biologie Moléculaire et Cellulaire (IGBMC), Université de Strasbourg, Illkirch, France; ^2^Centre National de la Recherche Scientifique (CNRS), Institut des Neurosciences Cellulaires et Intégratives (INCI), Université de Strasbourg, Strasbourg, France; ^3^Centre National de la Recherche Scientifique (CNRS), Institut de la Santé et de la Recherche Médicale (INSERM), CELPHEDIA-PHENOMIN-Institut Clinique de la Souris, (PHENOMIN-ICS), Université de Strasbourg, Illkirch, France; ^4^Centre National de la Recherche Scientifique (CNRS) UMR 7242, Université de Strasbourg, Illkirch, France

**Keywords:** *SCN9A*, sodium channel, nociception, pain, spontaneous pain, data analysis, statistical modeling, gdaphen

## Abstract

The voltage-gated sodium channel Nav1.7 is encoded by *SCN9A* gene and plays a critical role in pain sensitivity. Several *SCN9A* gain-of-function (GOF) mutations have been found in patients with small fiber neuropathy (SFN) having chronic pain, including the R185H mutation. However, for most of these variants, their involvement in pain phenotype still needs to be experimentally elucidated. In order to delineate the impact of R185H mutation on pain sensitivity, we have established the *Scn9a*^*R*185*H*^ mutant mouse model using the CRISPR/Cas9 technology. The *Scn9a*^*R*185*H*^ mutant mice show no cellular alteration in the dorsal root ganglia (DRG) containing cell bodies of sensory neurons and no alteration of growth or global health state. Heterozygous and homozygous animals of both sexes were investigated for pain sensitivity. The mutant mice were more sensitive than the wild-type mice in the tail flick and hot plate tests, acetone, and von Frey tests for sensitivity to heat, cold, and touch, respectively, although with sexual dimorphic effects. The newly developed bioinformatic pipeline, Gdaphen is based on general linear model (GLM) and random forest (RF) classifiers as well as a multifactor analysis of mixed data and shows the qualitative and quantitative variables contributing the most to the pain phenotype. Using Gdaphen, tail flick, Hargreaves, hot plate, acetone, cold plate, and von Frey tests, sex and genotype were found to be contributing most to the pain phenotype. Importantly, the mutant animals displayed spontaneous pain as assessed in the conditioned place preference (CPP) assay. Altogether, our results indicate that *Scn9a*^*R*185*H*^ mice show a pain phenotype, suggesting that the *SCN9A*^*R*185*H*^ mutation identified in patients with SFN having chronic pain contributes to their symptoms. Therefore, we provide genetic evidence for the fact that this mutation in Nav1.7 channel plays an important role in nociception and in the pain experienced by patients with SFN who have this mutation. These findings should aid in exploring further pain treatments based on the Nav1.7 channel.

## Introduction

Painful small fiber neuropathy (SFN) is a disorder of Aδ-fibers and C-fibers characterized by neuropathic pain symptoms and autonomic complaints. Several common diseases, such as diabetes mellitus and HIV have been reported to complicate SFN (Themistocleous et al., 2014; Cazzato and Lauria, 2017). The Nav1.7 channel is a voltage-gated sodium channel that plays a critical role in the generation and conduction of action potentials and is thus important for electrical signaling exhibited by the most excitable cells. It is preferentially expressed in the peripheral nervous system within sensory dorsal root ganglia (DRG) and sympathetic ganglia neurons (Black et al., 2012; Kanellopoulos et al., 2018; Cummins et al., 2020). Interestingly, gain-of-function (GOF) mutations in the *SCN9A* gene encoding for the α-subunit of Nav1.7 sodium channel have been identified in 5% of patients with painful idiopathic SFN (Waxman et al., 2014; Eijkenboom et al., 2019). However, the phenotype of these patients is complex and the impact of Nav1.7 channel mutations on these patients remains to be clarified. Faber et al. (2012) found the heterozygous c.554G > A, p.R185H mutation in the *SCN9A* gene of two unrelated patients with painful SFN (Faber et al., 2012). This mutation resulted in the hyperexcitability of rat DRG neurons when transfected into these neurons (Han et al., 2012). Later, the same mutation was also found to be associated with painful diabetic peripheral neuropathy (Blesneac et al., 2018). On the opposite, *SCN9A* loss-of-function bi-allelic mutations are known to result in congenital insensitivity to pain (CIP) while the mono-allelic carriers have normal pain sensitivity (Bennett et al., 2019; McDermott et al., 2019).

In order to explore the Nav1.7-R185H genotype-phenotype association and the underlying mechanisms for Nav1.7-R185H sodium channel mutation in idiopathic SFN, we have created a *Scn9a*^*R*185*H*^ mouse model and characterized this model for nociceptive behavior. This mouse line was generated using the CRISPR/Cas9 technology. We found that *Scn9a*^*R*185*H*^ mRNA and protein were both expressed at comparable levels in the mutant mice as wild-type (wt) control mice. Pain sensitivity of the mutant mouse line was investigated using behavioral tests of sensitivity to thermal and mechanical stimuli and in an ongoing spontaneous pain model. As sex is known to be an important variable in pain (Sadler et al., 2022), pain behavior was evaluated in both sexes. Our results indicate that the *Scn9a*^*R*185*H*^ mice show a pain phenotype, suggesting that the *SCN9A*^*R*185*H*^ mutation identified in patients with SFN contributes to their pain symptoms. Therefore, altogether we provide genetic evidence for the fact that the *SCN9A*-encoded Nav1.7 channel plays a crucial role in pain behavior.

## Materials and Methods

### Animals

#### Experimental Subjects and Ethical Approval

Animal experiments were performed in agreement with the EC directive, 2010/63/UE86/609/CEE and in compliance with the animal Welfare policies of the French Ministry of Agriculture (law 87 848) and with protocols approved by local ethical committees (Com’Eth, « Comité d’Ethique pour l’Expérimentation Animale IGBMC-ICS », with the agreement number 20880; or CREMEAS). C57BL/6NCrl mice were used for the mutant line generation, and C57BL/6NCrl was the genetic background throughout the study. The number of mice that were used followed the 3R principles. Mice were bred at the ICS animal facility and the behavioral experiments were performed at «Institut de Génétique et de Biologie Moléculaire et Cellulaire» (IGBMC), at the « Chronobiotron UMS3415 » and at « Institut des Neurosciences Cellulaires et Intégratives » (INCI) (see Section below). The mice were housed under a 12-h/12-h light/dark cycle (lights on at 7 a.m.) and at 21 ± 1°C, 55 ± 10% humidity condition. Food and autoclaved tap water were available *ad libitum*. Each cage housed 2–5 mice. All mice were habituated to the experimental environment.

#### Establishment of the Genetic Animal Model

**FIGURE 1:**
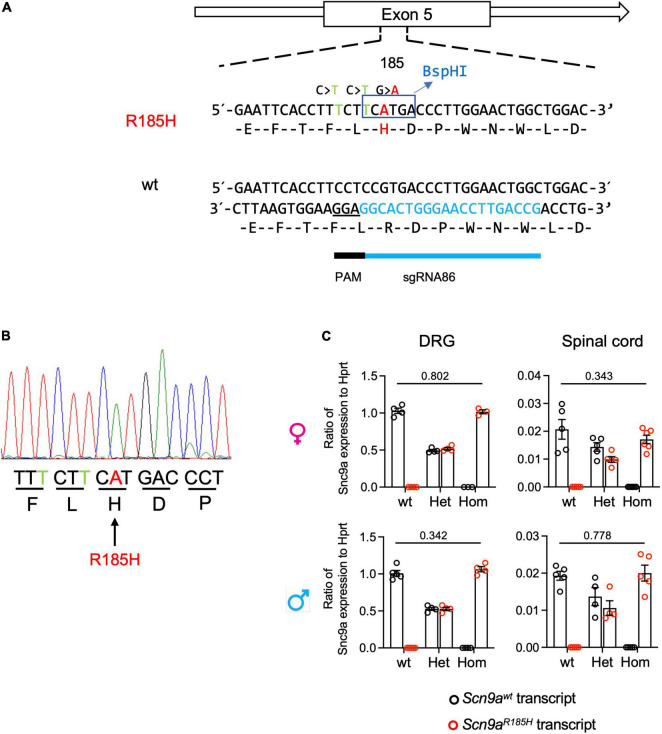
Generation of *Scn9a*^*R*185*H*^ mutant mice and *Scn9a* transcript expression in wild-type (wt) and mutant mice. **(A)** Scheme for *Scn9a* targeting strategy. The sgRNA86 guide RNA and protospacer adjacent motif (PAM) are shown. The ssODN contained three basic mutations including two silent mutations and one-point mutation. The first silence mutation, C > T was PAM mutation designed to avoid Cas9 recut. The second mutation, C > T, was designed for *Bsp*HI enzyme restriction site for genotyping. The third G > A mutation was designed to get the R185H found in human patients. **(B)** Mutant allele sequence characterized through Sanger sequencing of F0 founder DNA. The silence-mutated nucleotides are indicated in green and targeted point mutant nucleotide in red. **(C)** *Scn9a* micro-RNA (mRNA) expression in dorsal root ganglia (DRG) and spinal cord, of wt and *Scn9a*^*R*185*H*^ mutant mice of both sexes. There was no significant effect of genotype. DRG. females: wt *n* = 4; Het *n* = 4; Homo *n* = 3; males: wt *n* = 5; Het *n* = 4; Homo *n* = 4; Spinal cord. Females: wt *n* = 5; Het *n* = 5; Homo *n* = 6; males: wt *n* = 5; Het *n* = 4; Homo *n* = 5. Scn9a transcript expression was normalized to *Hprt* expression. Results are shown as means ± SEM. Two-tailed unpaired *t*-test for wt allele transcript expression in wt mice vs. mutant allele transcript expression in homozygous mutant mice. Refer to Supplementary Table 5 for statistics.

The *Scn9a*^*R*185*H*^ mouse line was generated by using the CRISPR/Cas9 system. The CRISPOR online software was used to select high specificity-score sgRNAs with a low number of predicted off-target sequences. A single-stranded oligonucleotide was used that contained the CGT to CAT change encoding for the R185H mutation with 2 additional silent mutations, one to mutate the PAM sequence and the other to generate a new *Bsp*HI diagnostic restriction site (Figure 1A). Before injecting sgRNAs into eggs, the efficiency of sgRNA was tested *in vitro* on the targeted DNA by polymerase chain reaction (PCR). After checking sgRNA efficiency *in vitro*, sgRNA, Cas9, and ssODN were microinjected into the cytoplasm and into the pronucleus of the fertilized oocytes of C57BL/6NCrl. The surviving embryos were implanted into the oviducts of pseudo-pregnant CD1 mice. Resulting pups were screened by PCR followed by *Bsp*HI digestion to detect the expected mutation (R185H) and T7 endonuclease to detect indels. Potential founders were identified and were confirmed by Sanger sequencing. Selected founders were bred with wt C57BL/6NCrl mice to generate F1 heterozygous animal. All F1 mice were analyzed for their confirmed genotype by Sanger Sequencing to establish the *Scn9a*^*R*185*H*^ mouse line. These mice were also checked for off-target effects, by using the PCR primers described in Supplementary Table 1. Thereafter, the F1 founder (53-11) was backcrossed with C57BL/6NCrl wt mice for four generations to generate the *Scn9a*^*R*185*H*^ mutant line containing mutant and wt mice of both sexes for further experiments. Finally, heterozygous *Scn9a*^+/*R*185*H*^ males and females were crossed to generate *Scn9a*^+/+^ (wt), heterozygous *Scn9a*^+/R185*H*^ (het), and homozygous *Scn9*^*R*185*H/R*185*H*^ (homo) mice. This breeding strategy led to 24.2% wt, 50.5% het, and 25.3% homo individuals.

#### Determination of Genotype

Genotype was determined from tail biopsies on 10-day old pups. Crude genomic DNA was extracted by DirectPCR Lysis Reagent-Tail (Viagen Biotech, Cat # 101-T) according to the manufacturer’s instructions. Phusion Hot Start II High-Fidelity DNA polymerase kit (Thermo Scientific) was used. The PCR reaction contained 5 μl of diluted genomic DNA, 4 μl of 5 × Phusion HF Buffer, 0.4 μl of dNTP mix (dATP, dCTP, dGTP, and dTTP at 10 mM, Thermo Scientific), 2 μl of each primer at 0.5 nM, and 0.2 μl of Phusion polymerase and H_2_O in a total volume of 20 ml. PCRs were done under the following conditions: 98°C for 30 s followed by 30 cycles of 98°C for 8 s, 62°C for 30 s, and 72°C for 1 min and a final elongation step for 5 min at 72°C in a T100 thermocycler (Bio-Rad). Ten microliters of PCR product were then digested in a final volume of 20 μl added to 0.2 μl of *Bsp*HI enzyme and 2 μl of enzyme buffer. PCR outcome was analyzed on a 2% agarose gel. Information about primer sequences is shown in Supplementary Table 2.

### Determination of Transcript Expression by Reverse Transcriptase Digital Droplet Polymerase Chain Reaction

The DRGs from both sides, the spinal cord, and half brain, were collected from the mutant and wt mice and flash-frozen in liquid nitrogen. All samples were disrupted with Precellys^®^ CK14 Lysing Kit in TRIzol Reagent, and the total RNA was purified using the RNeasy Fibrous Tissue Mini Kit (Qiagen) according to the manufacturer’s instructions. The quality of the samples was checked using an Agilent 2100 Bioanalyzer (Agilent Technologies). Complementary DNA (cDNA) synthesis was performed using the SuperScript™ VILO™ cDNA Synthesis Kit (Invitrogen). Digital droplet PCR (ddPCR) was performed in 20 μL reactions containing 1 × ddPCR Supermix for probes (No dUTP), 250 nM of each probe, 900 nM of specific primers, and 50 ng of DNA according to the manufacturer’s recommendations (PCR conditions: 10 min at 95°C, 40 cycles of 30 s at 94°C, 30 s at 55°C, and 10 min at 98°C), in a QX200 Droplet Digital PCR System (Bio-Rad) and analyzed by QuantaSoft Software (Bio-Rad). Sequences of probes and primers are shown in Supplementary Table 3.

### Histological Analysis

#### Tissue Preparation

Mice were anesthetized with ketamine/xylazine and perfused intracardially with 40 ml of phosphate buffer saline (PBS) with 0.1 M pH 7.4 followed by 4% of paraformaldehyde (PFA) in PBS with 0.1 M pH 7.4. Both sides of L4 to L6 lumbar DRG and sciatic nerve samples were dissected out and post-fixed for overnight at 4°C in 4% of PFA in PBS, thereafter cryoprotected at 4°C in 30% of sucrose in PB for 3–7 days, embedded in optimal cutting temperature (OCT) medium, frozen, and kept at −80°C. DRG (8 μm thick) and sciatic nerves (10 μm thick) were longitudinally cut with a cryostat and kept at −20°C.

#### Immunohistochemistry

Light antigen retrieval was performed in 0.1 M of PB with pH 7.3, 0.2% of hydrogen peroxide, and 0.05% of Triton X-100 for 25 min at room temperature (RT). Sections were then washed twice in PBS and incubated in a blocking solution of tris-buffered saline (TBS) with 0.05% of Tween 20, 2% of bovine serum albumin (BSA), and 2% of normal donkey serum (NDS) for 1 h before applying primary antibodies overnight at 4°C. The following primary antibodies (diluted in the blocking solution) were used: Rabbit anti-SCN9A-ATTO Fluor-663 antibody (1:100, ASC-008-FR, Alomone Labs, Jerusalem, Israel), Mouse anti-PGP9.5 antibody (1:200, ab8189, Abcam, Cambridge, United Kingdom), and rabbit anti-PGP9.5 (1:500, ab108986, Abcam). For the detection of the primary antibody, secondary antibody raised in donkey and conjugated with Alexa-647 fluorophore was used (1:500, Molecular Probes, Fisher Scientific, Illkirch, France) for 1 h at RT. DAPI staining (1:2000, Molecular Probes, Thermofisher, Illkirch, France) was performed at the same time as in the secondary antibody. Sections were then washed twice with PBS then placed on superfrost glass slides. One drop of Immu-Mount (Fisher Scientific, Illkirch, France) was added over the tissue sections and a coverglass was placed over the slide.

#### Image Acquisition and Analysis

Dorsal root ganglia and sciatic nerve images were acquired with the Leica fluorescence microscope using a 20 × (DRG) and 40 × (sciatic nerves) dry objective, the 20 × and 40 × resolution were achieved with a digital zoom factor. Image acquisitions in the sequential mode (single excitation beams: 405, 488, and 633 nm) were used for marker co-localization to avoid potential crosstalk between the different fluorescence emissions. Images were acquired with the LCS (Leica) software using randomly selected sections. The ImageJ software cell counter (approximately 4 non-adjacent sections per condition and per animal) was used to count on-screen neurons expressing a given fluorescent marker manually and blindly. Only neurons from L4 to L6 DRGs with a visible nucleus were considered. Cells expressing a given fluorescence were analyzed separately. During the analysis, we recorded all cross-sectional areas of cell profiles for Nav1.7 positive and negative neurons in PGP9.5-positive total neurons, that were categorized to different sizes based on their area. Sciatic nerves were analyzed for fluorescence density by using the same Nav1.7 and PGP9.5 antibodies, by applying a threshold of fluorescence detection. The mean of fluorescence density (total fluorescence density/area) was calculated to determine the Nav1.7 fluorescence.

### Behavioral Characterization

A series of behavioral experiments were conducted on mutants and wt littermates in order to evaluate the nociceptive behavior and motor condition. All behavioral tests were done between 9:00 a.m. and 5:00 p.m. Animals were transferred to the experimental room 30 min before each experimental test. The behavioral tests were performed in the following order: String test, crenelated bar, von Frey, Hargreaves Plantar, tail flick, tail pressure, hot plate, acetone test, cold plate, and odor habituation and discrimination. Mice were tested at 2-month-age. Between two nociceptive tests, there was a gap of at least 2 days. Both females and males of the different genotypes were analyzed. The behavioral analysis was done with an experimenter who was unaware of the genotype of the mouse.

#### String Test

The grip string test (home-made) was used to measure the muscle strength. The equipment used was a wire stretched horizontally 40 cm above a table. The time required for a forelimb-hanging mouse to gain hindlimb traction as latency was measured, with 20 s cutoffs. Three consecutive trials were done by 5 min intervals.

#### Crenelated Bar

The notched/crenelated bar test was used for motor coordination and balance. The method described by Carter et al. (2001) was used. Briefly, mice were kept on an elevated crenelated bar and they had to traverse a distance from far away to a distance closer to the home cage. Thereafter, the time to traverse the whole crenelated bar were recorded.

#### Odor Habituation and Discrimination

This test is adapted from the one described by Duchon et al. (2011). Briefly, mice were placed 5 times for 2 min in a small cage (22 cm × 15 cm). Perforated tubes containing a small piece of Whatman paper soaked with orange flower water were placed in the center of each cage. The time of sniffing was recorded on six trials of 2 min each with the orange flower extract. Thereafter, the orange flower extract was replaced by vanilla extract for the last trial. Inter-trial time ranged from 8 to 10 min. The time spent on sniffing the odors was recorded.

#### Hot Plate

Hot plate response refers to the method reported by Huang et al. (2019). Mice were habituated on the plate (reference BIO-HC1.00, Bioseb, Vitrolles, France) at RT one day before the test. Mice were placed on the plate at 48, 52, and 56°C with respective cutoff times to avoid tissue damage: 2 min for 48°C, 1 min for 52°C, and 30 s for 56°C. The latency to the first hindlimb reaction and to jump as well as the coping reactions (flicking, licking, and jumping) were recorded until cutoff.

#### Tail Flick

The tail flick test was performed at Panlab (L7306, Panlab, Bioseb, Vitrolles, France) as described by Weibel et al. (2013). Mice were habituated for 30 min in the procedure room prior to test. During the test, mice were wrapped into 50 ml tube with the whole tail exposed. Three different distances from the tail tip were tested for the reaction to the light beam. The mean of these three measurements of latency was calculated.

#### Hargreaves Plantar

The Hargreaves Plantar test (Bioseb, Vitrolles, France) was used to quantify the responses of mice to noxious heat at the hind paw by applying a radiant infrared heat stimulus (Weibel et al., 2013). Before the test, the mice were habituated to the glass plate in bottom-opened transparent plexiglass boxes (7 × 9 × 7 cm) so that withdrawal latencies could be clearly determined, usually taking 30 to 45 min. A radiant heat beam was positioned underneath the paws of the mice. The withdrawal latencies were recorded for both right and left hind paws. The time between testing the first and the second paw was at least 5 min. For each mouse, the response assays were tested for a maximum of 4 times. The means of all measurements of both hind paws were calculated.

#### Thermal Gradient Ring

The analysis of thermal preference in mice was performed with the thermal gradient ring (UgoBasile, Gemonio, Italy) (Touska et al., 2016) at INCI. All mice were adapted to the equipment on day 1 with the ring adjusted to RT for 30 min. Mice were recorded on day 2 for 4 consecutive periods of 15 min for a total of 60 min using the 15–40°C ring gradient. Behavior was videotaped with camera and analyzed with the ANY-maze Software (V5, UgoBasile. Gemonio, Italy). Temperature preference was calculated as the percent of time spent above the surface at a given temperature. Temperature preference was averaged for each 15-min period. The temperature preference over the first 30 min was calculated as the mean of temperature preference from the first two 15-min periods.

#### Acetone Test

The acetone test was performed to evaluate the behavioral response to cooling as described by Deuis et al. (2017). Mice were placed and habituated in bottom-opened transparent plexiglass boxes (7 × 9 × 7 cm) on a mesh grid floor. One day before the test, the mice were habituated to the boxes for 1 h. On the day of testing, the mice were allowed to habituate for 30 min and then 10 μL of acetone was applied to the center of the plantar surface of each hind paw. Acetone was applied on three successive testing sessions for each paw. The interval between each application was at least 5 min. The number of flicking and licking of affected paw were counted for 30 s after acetone application. The duration of these paw reactions was also recorded.

#### Cold Plate

The cold plate assay (Bioseb, Vitrolles, France) was used to assess the sensitivity of the mice to the cold 5°C temperature as described by Reiss et al. (2021). One day before the test, the animals were habituated to the plate at RT in transparent plastic boxes as described above. A 5-min cutoff time was used. The number of hind paw lifts and their duration were recorded on each paw and the mean per animal was calculated.

#### Von Frey Test

The evaluation of sensitivity to touch by the von Frey test was performed on different cohorts at IGBMC (I) or INCI (II). At IGBMC, mice were placed in the same transparent plastic boxes and habituated as described for the acetone test. A series of eight von Frey filaments (with the bending force of 0.008 to 2 g) (Bioseb, Vitrolles, France) was applied to the hind paw using the up-and-down method. Each paw was scored on two successive testing sessions. The withdrawal threshold was calculated by the Excel program generously provided by A. Basbaum (University of California San Francisco). The mean of the sensitivity of the two hind paws was calculated. At INCI, the mechanical threshold of hind paw withdrawal was evaluated using von Frey hairs (Aniphy Vivo-Tech, Salon-de-Provence, France). Mice were placed in clear Plexiglas boxes (7 × 9 × 7 cm) on an elevated mesh screen. After a habituation time of around 10 min, the filaments were applied to the plantar surface of each hind paw in a series of ascending forces (0.6 to 8 grams). Each filament was applied five times per paw, being applied until it just bent, and the threshold was defined as 3 or more withdrawals observed out of the 5 trials. The mean of the sensitivity of the two hind paws was calculated.

#### Tail Pressure

A gradually increasing pressure was applied to the tail using the equipment Pressure AnalgesiMeter (Ugo Basile, Italy) as previously done by Weibel et al. (2013). The tail withdrawal threshold was recorded for each mouse in three successive testing sessions, with a 500 g cutoff.

#### Conditioned Place Preference

All experiments were conducted by using the single trial conditioned place preference (CPP) protocol at INCI as previously described by Barthas et al. (2015). The apparatus (Imetronic, Pessac, France) consists of 3 Plexiglas chambers separated by manually operated doors. Two chambers (size 15 cm × 24 cm × 33 cm) distinguished by the texture of the floor and by the wall patterns are connected by a central chamber (size 15 cm × 11 cm × 33 cm). On the first, second, and third days (pre-conditioning), the animals were free to explore the apparatus during 30 min, and the time spent in each chamber was recorded to control for the lack of spontaneous preference for one compartment. Animals spending more than 75% or less than 25% of the total time in one chamber were excluded from the study. On the 4th day (conditioning), the animals were intrathecally injected with either saline solution (10 μL in the morning) or clonidine solution (10 μL in the afternoon) and restricted in one chamber during 15 min, switching the chamber between the morning and the afternoon. The delay between the two injections was 4 h. On the 5th day, the animals were free to explore the 3 chambers and the time spent in each chamber was recorded for 30 min.

#### Dark-Light Test

The anxiety-like behavior was evaluated with the dark-light test (Vogt et al., 2016) at INCI with a two-compartment testing box (18 cm × 18 cm × 14.5 cm). One compartment was brightly illuminated (1,500 lux), whereas the other was dark. Mice were placed in the dark compartment at the beginning of the test, and the time spent in the lit compartment was recorded for 5 min.

#### Forced Wimming Test

This test was performed at INCI to evaluate despair-like behavior (Porsolt et al., 1977). The mice were lowered into a glass cylinder (height 17.5 cm, diameter 12.5 cm) containing 11.5 cm of water (23–25°C). The test duration was 6 min, and since only little immobility is usually observed during the first 2 min, the duration of immobility was quantified during the last 4 min of the test. The mice were considered to be immobile when they floated upright in the water, with only minor movements to keep its head above the water.

### Gdaphen Analysis for the Identification of the Variables Contributing the Most to the Genotype or Sex Discrimination

The Gdaphen R pipeline was applied for the identification of the major variables contributing to the genotype or sex discrimination as described previously by Chidiac et al. (2021). Gdaphen is a public R package (Muniz et al., available on github https://github.com/munizmom) developed to identify the explanatory variables from the experimental data. We considered 18 variables, genotype, and sex as well as the following 16 behavioral variables: hot plate 48°C latency to the first response (s), hot plate 48°C latency to jump (s), hot plate 48°C coping reactions (nb), hot plate 52°C latency to the first response (s), hot plate 52°C latency to jump (s), hot plate 52°C coping reactions (nb), hot plate 56°C latency to the first response (s), hot plate 56°C latency to jump (s), hot plate 56°C coping reactions (nb), tail-flick latency (s), Hargreaves latency (s), acetone duration of paw reactions (s), acetone paw reactions (nb), cold plate 5°C paw lifts (nb), von Frey threshold (g), and tail pressure threshold (g). The analysis included the identification of the variables contributing the most to the genotype or sex discrimination by using two classifiers, the supervised algorithm generalized linear model (GLM) and the unsupervised algorithm, random forest (RF). The weight of each individual variable and the overall weight for each test was predicted and visualized by using multiple factor analysis (MFA).

### Statistical Analysis

Results are expressed as means ± SEM. Statistical analyses were performed using GraphPad Prism 9 software. Results were analyzed using two-tailed unpaired Student’s *t*-test to assess differences between two groups for normally distributed data or by Mann–Whitney test if the data did not pass the normality test. Genotype and age effects, and genotype and sex effects were analyzed using two-way ANOVA. The genotype effect in each sex was analyzed by one-way ANOVA followed by Dunnett’s multiple comparison test, when appropriate. A *P*-value less than 0.05 was considered as statistically significant. The detailed statistics are presented in Supplementary Tables 6–14. *P*-values for genotype differences are shown in figures when significant or close to significance.

## Results

### Generation and Characterization of *Scn9a*^*R*185*H*^ Mutant Mice

The *Scn9a*^*R*185*H*^ mouse line was generated by using the CRISPR/Cas9 technology. After checking sgRNA validity *in vitro*, different concentrations of sgRNA, Cas9, and ssODN were microinjected into eggs. Sixty F0 mice born were screened by PCR. The different product sizes found by gel analysis suggested a variety of alleles that were confirmed by Sanger sequencing (refer to Supplementary Figure 1). Several F0 individuals showed point mutation and high mosaicism in both alleles. Finally, five potential F0 founders were used to cross with C57BL/6NCrl wt mice to generate F1 founders. We successfully got germline transmission to the F1 generation (Figure 1B). We selected the F1 animal 53-11 to establish the *Scn9a*^*R*185*H*^ mouse line. There has been much concern raised in the scientific community over the specificity of the CRISPR/Cas9 system. Therefore, we analyzed several predicted off-target sites that were based on the number of mismatches to the target sequences and the homologous genes by the CRISPR design tool.^1^ No evidence of Cas9-mediated deletions or base-pair changes in any of the seven mostly predicted off-target sites (Supplementary Table 4) was found after comparing with wt sequences in Ensembl genome database^2^ (Supplementary Figure 2). Thereafter, the heterozygous F1 (53-11) was used as the founder to generate the *Scn9a*^*R*185*H*^ mutant line.

When evaluating *Scn9a* transcript expression in the *Scn9a*^*R*185*H*^ mice, the two-way ANOVA showed a strong effect of genotype, and there was no effect of sex on expression in both the DRG and spinal cord. *Scn9a^wt^* and *Scn9a*^*R*185*H*^ transcripts were equally expressed in the DRG and spinal cord of wt and homozygous mutant mice, respectively, and in both sexes (Figure 1C and Supplementary Table 5). Heterozygous animals expressed 50% wt and 50% mutant transcript alleles. Globally, these results show that *Scn9* transcripts are expressed equally in the mutant and wt mice, suggesting that the *Scn9a*^*R*185*H*^ mutation does not impact on *Scn9a* transcript expression.

### Nav1.7 Protein Expression in Dorsal Root Ganglia and Sciatic Nerves of *Scn9a*^*R*185*H*^ Mice

**FIGURE 2:**
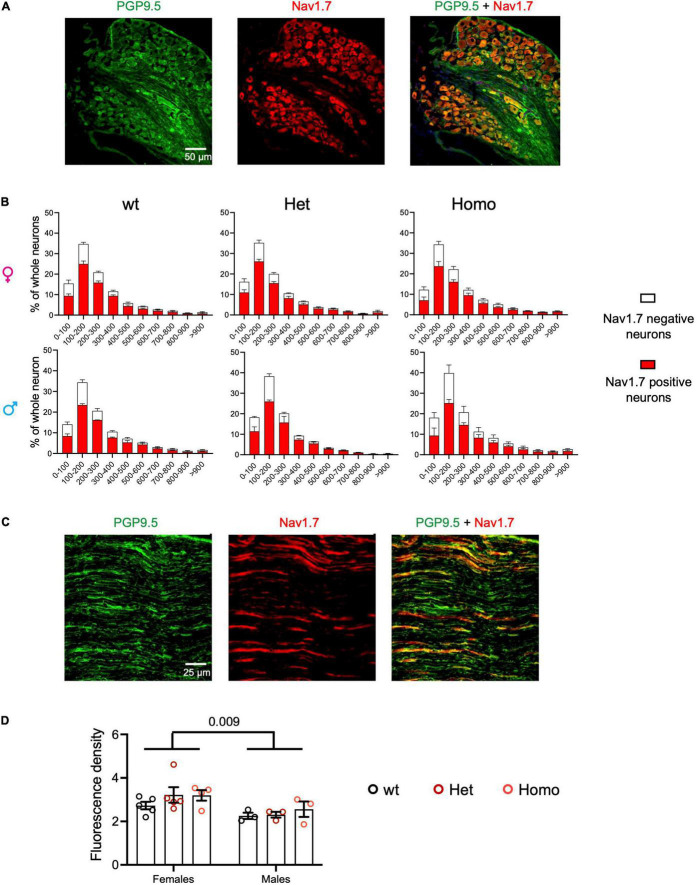
Nav1.7 protein expression of DRG and sciatic nerve in *Scn9a*^*R*185*H*^ mutant mice. **(A)** Representative images of PGP9.5 (green) and Nav1.7 (red) fluorescent immunostaining for all neurons (PGP9.5-positive) and Nav1.7-positive neurons in lumbar DRG of wt animals. Scale bar 50 μm. **(B)** Size distribution for Nav1.7-positive (red) and negative (white) neuron cross-sectional areas in *wt*, het, and homo female and male mice. Females: *n* = 5/group; male: *n* = 3/group. Results are shown as means ± SEM. The two-way ANOVAs for genotype and neuron size effects in each sex showed a size effect and no genotype effect. **(C)** Representative images of fluorescent PGP9.5 (green) and Nav1.7 (red) immunostaining of sciatic nerves of *wt* animals. Scale bar of 25 μm. **(D)** Mean fluorescence density Nav1.7 immunostaining of sciatic nerves in wt, het, and homo female and male mice. Females, *n* = 4–5/group; males, *n* = 3/group. Results are shown as means ± SEM. The two-way ANOVA for genotype and sex indicated a sex effect and no genotype effect. The *P* value of 0.009 is for sex effect. See Supplementary Table 6 for statistics.

The impact of the *Scn9a*^*R*185*H*^ mutation on NA_*V*_1.7/SCN9A protein expression was analyzed in the DRG and sciatic nerves by immunofluorescent staining of Nav1.7, together with labeling neurons with PGP9.5. The SCN9A protein was found expressed in 78.4 ± 3.4% of neurons in wt and mutant DRGs and with a comparable profile according to cell-size distribution (Figures 2A,B and Supplementary Table 6). The size distribution of all DRG neurons was also similar in the three genotypes, with most neurons of small and medium size, as expected. No genotype difference was detected (Supplementary Figure 3 and Supplementary Table 6), indicating that the mutation did not impact on neuron size distribution. Sciatic nerves were analyzed for fluorescence density of Nav1.7 and of PGP9.5 protein as a nerve marker (Figure 2C) and Nav1.7 density was compared in wt and mutant mice of both sexes. The two-way ANOVA showed a sex effect and no genotype effect, with females expressing globally more Nav1.7 in sciatic nerves than males (Figure 2D and Supplementary Table 6). Overall, these results indicate that Nav1.7 protein is expressed similarly in mutant and wt mice, suggesting that the *Scn9a*-R185H mutation does not alter Nav1.7 protein expression.

### Normal Body Weight and Motor Function in *Scn9a*^*R*185*H*^ Mice

First, as *Scn9a* deletion in hypothalamic neurons was reported to disrupt body weight regulation (Branco et al., 2016), we measured body weight in mutant mice on a weekly basis from week 1 to week 31 of age. There was no difference between mutant and wt animals for body weight (Supplementary Figure 4A). To determine whether muscle strength and motor coordination were affected in the mutant line, string test and crenelated bar tests were performed. As shown by two-way ANOVA, there was no effect of genotype and sex for both string test and crenelated bar test. Therefore, no major signs of behavioral abnormalities were found in mutant mice in these tests (Supplementary Figure 4B). Previous studies on *Scn9a* loss-of-function models showed that Nav1.7 was necessary for pain sensation (Xue et al., 2021) and was also an essential requirement for odor perception in both mice and humans (Weiss et al., 2011). We then assessed the olfactory function using an odor habituation and discrimination test in mutant mice. We found that the homozygous females had lower sniffing time in the first trial as compared to wt controls while all other parameters including the response of a new olfactory stimulus were comparable in mutant and wt animals, indicating that *Scn9a*^*R*185*H*^ mutants had a normal odor discrimination capacity (Supplementary Figure 4C).

### Enhanced Heat Pain Sensitivity in *Scn9a*^*R*185*H*^ Mice

**FIGURE 3:**
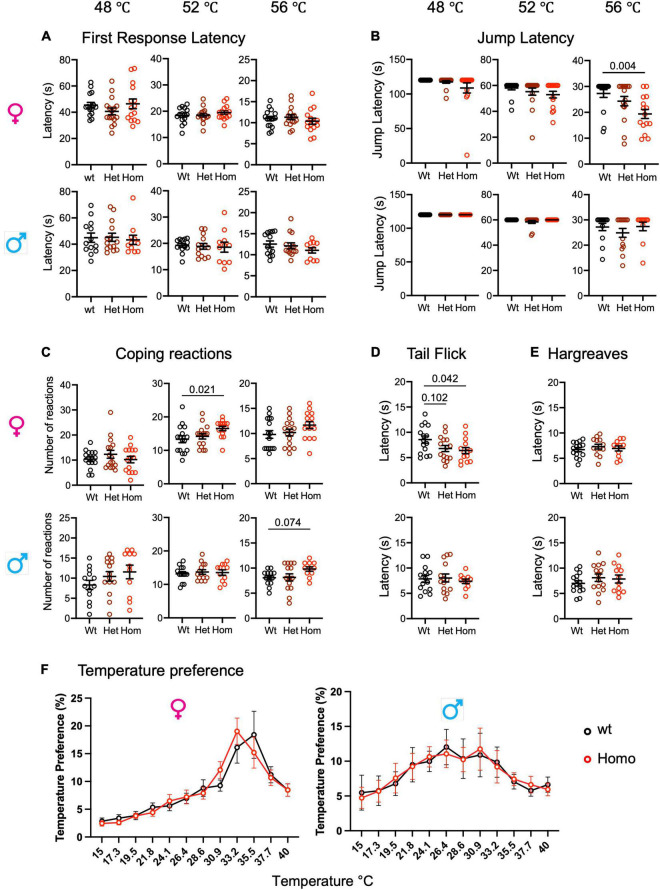
Enhanced heat pain sensitivity in *Scn9a*^*R*185*H*^ mice. **(A)** Wild-type and *Scn9a*^*R*185*H*^ mice showed comparable first response latency on the hot plate. **(B)** Homozygous *Scn9a*^*R*185*H*^ females had decreased jump latency in 56°C Hot Plate. **(C)** Homozygous *Scn9a*^*R*185*H*^ female mice showed more coping reactions on the 52°C hot plate. **(D)** Heterozygous and homozygous *Scn9a*^*R*185*H*^ females had lower tail latency in the tail flick test. **(E)** No difference was found between wt and mutant mice in the Hargreaves test. **(F)** Control wt and mutant mice showed the same temperature preference. Results are shown as means ± SEM. Hot plate, females: wt, *n* = 16; Het, *n* = 16; Homo, *n* = 15; males: wt, *n* = 13; Het, *n* = 14; Homo, *n* = 11. Tail flick: females: wt, *n* = 15; Het, *n* = 15; Homo, *n* = 14; males: wt, *n* = 14; Het, *n* = 14; Homo, *n* = 11. Hargreaves: females: wt, *n* = 14; Het, *n* = 13; Homo, *n* = 11; males: wt, *n* = 14; Het, *n* = 14; Homo, *n* = 11. Temperature preference: females: wt, *n* = 12; Homo, *n* = 12; males: wt, *n* = 11; Homo, *n* = 11. One-way ANOVA on females and males and Dunnett’s multiple comparison tests for hot plate, tail flick, and Hargreaves tests; two-way ANOVA for the thermal preference ring test. *P*-values for genotype difference are shown when significant or close to significance. See Supplementary Tables 7–9 for statistics.

The two heterozygous patients with R185H having chronic pain were a man and a woman, respectively, who had abnormal warm pain sensation even when they were 23–24-year-old (Faber et al., 2012). To investigate whether the R185H mutation alters heat pain sensitivity in mutant mice, we tested mutants of both sexes on the hot plate at 48, 52, and 56°C, respectively, as well as in the tail flick, Hargreaves plantar tests, and thermal preference tests (Figure 3). First, on the hot plate, there was no genotype or sex effect for the first response latencies at the three temperatures (Figure 3A and Supplementary Table 7). We also analyzed the jump latencies of the *Scn9a*^*R*185*H*^ line. The two-way ANOVA revealed an effect of sex at 52°C and an effect of sex as well as an interaction between sex and genotype at 56°C. At this temperature, the homozygous females had shorter jump latency as compared to their wt counterparts (Figure 3B and Supplementary Table 7). Until recently, only the latency to the first hind paw response or to jump were used as outcomes for the hot plate test. Still, in 2019, it was found that mutant mice lacking spinal preprotachykinin-positive neurons had normal first response latency on the hot plate but had reduced coping reactions (Huang et al., 2019), indicating that defensive reactions that limit injury may constitute valuable measures of pain. Therefore, we scored coping reactions (number of flicks, licks, and jumps) in addition to the first response and jump latencies. At 48°C, there was no effect of genotype or sex in the two-way ANOVA (Figure 3C and Supplementary Table 7). At 52°C, there was a trend for genotype effect as analyzed by two-way ANOVA, a genotype effect in females as shown by one-way ANOVA, and increased coping reactions in homozygous females. At 56°C, both genotype and sex effects were evidenced by two-way ANOVA, with a trend toward more coping reactions in homozygous males (Figure 3C and Supplementary Table 7). The mutant mice were also tested for their sensitivity to a radiant heat stimulus in the tail flick and Hargreaves plantar tests. In the tail flick test, the mutant females were found to be more sensitive than wt controls, with shorter reaction latencies (Figure 3D and Supplementary Table 8), while male mutants had no phenotype. In the Hargreaves test, no effect of genotype or sex was found (Figure 3E and Supplementary Table 8). Additionally, the mutant animals were evaluated in the thermal gradient ring, a novel device that allows for the analysis of thermal preference. Thermal preference was tested at non-noxious temperatures ranging from 15 to 40°C. In this setting, wt males showed a plateau-shaped temperature preference at 26.4–30.9°C which was comparable in male mutants, and wt females showed a peak-like preference for 35.5°C which did not statistically differ in mutant females (Figure 3F, Supplementary Figure 5, and Supplementary Table 9). Overall, the mutant animals, especially females were more sensitive to noxious heat in the tail flick test and in the hot plate assay at the highest temperatures for jump latency and coping reactions.

### Sensitivity to Cool and Cold Stimuli in *Scn9a*^*R*185*H*^ Mice

**FIGURE 4:**
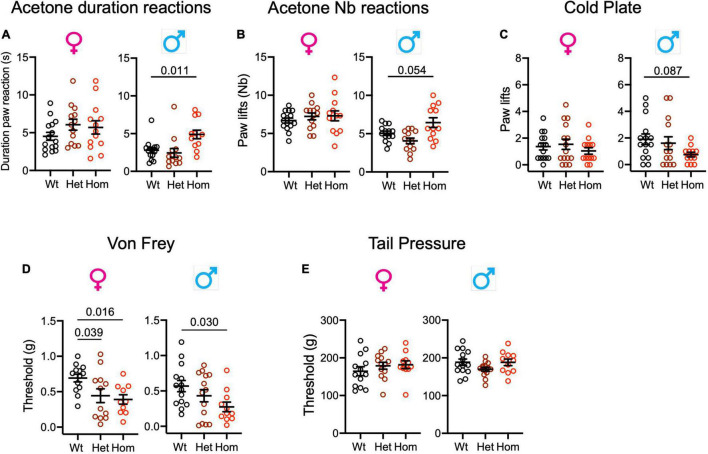
*Scn9a*^*R*185*H*^ mice show enhanced sensitivity to cooling and touch stimuli. **(A,B).** Homozygous mutant males show increased duration and number of paw reactions in the acetone test. **(C).** Male mutant mice show a trend to reduced number of paw lifts on the cold plate. **(D).** Mutant mice display lower sensitivity threshold in the von Frey test. **(E).** Mutant mice show no difference to wt mice in the tail pressure test. Results are shown as means ± SEM. Acetone duration of paw reactions and number of paw reactions: females: wt, *n* = 15; Het, *n* = 13; Homo, *n* = 13; males: wt, *n* = 14; Het, *n* = 13; Homo, *n* = 12. Cold plate: females: wt, *n* = 15; Het, *n* = 15; Homo, *n* = 13; males: wt, *n* = 15; Het, *n* = 14; Homo, *n* = 12. Von Frey: females: wt, *n* = 13; Het, *n* = 13; Homo, *n* = 10; males: wt, *n* = 14; Het, *n* = 14; Homo, *n* = 11. Tail pressure: females: wt, *n* = 13; Het, *n* = 13; Homo, *n* = 11; males: wt, *n* = 14; Het, *n* = 14; Homo, *n* = 11. One-way ANOVA on females and males and Dunnett’s multiple comparison tests. *P*-values for genotype difference are shown when significant or close to significance. See Supplementary Tables 10, 11 for statistics.

To determine whether *Scn9a*^*R*185*H*^ mutants had altered cold sensation, we performed the acetone and cold plate tests for assessing the reactions to cool (12∼15°C) and cold (5°C) stimuli. In the acetone test, the two-way ANOVA showed a strong effect of sex and an effect of genotype for both the number and the duration of paw reactions. The genotype effect was confirmed for males by one-way ANOVA on separate sexes (Supplementary Table 10A). As shown in Figures 4A,B, homozygous mutant males showed increased paw reaction duration as compared to wt controls indicating a hypersensitivity to cool temperatures. In the cold plate test, the two-way ANOVA for the number of paw lifts evidenced no sex effect and a trend for genotype effect which did not reach significance in the one-way ANOVA on separate sexes (Figure 4C and Supplementary Table 10B). Overall, the *Scn9a*^*R*185*H*^ mutant mice showed an increased sensitivity to cool temperatures.

### *Scn9a*^*R*185*H*^ Mice Are More Sensitive to Touch Stimuli

In order to evaluate mechanical sensitivity in *Scn9a* mutant mice, the von Frey and tail pressure tests were applied as models for touch and noxious mechanical stimuli. For the von Frey test, the two-way ANOVAs showed the effect of genotype, with both homo females and males showing enhanced sensitivity (Supplementary Figure 6A and Supplementary Table 11). In the tail pressure test, wt and *Scn9a*^*R*185*H*^ mice had similar sensitivity thresholds (Figure 4E and Supplementary Table 11).

### Gdaphen Analysis for the Identification of the Variables Contributing the Most to the Genotype or Sex Discrimination

**FIGURE 5:**
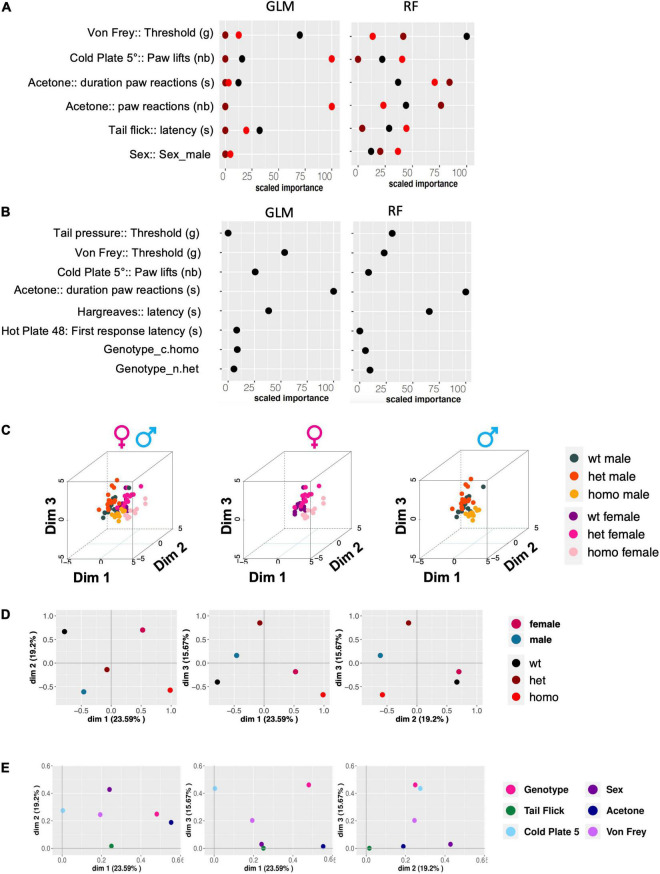
Gdaphen analyses to identify the variables contributing the most to genotype and sex discrimination. **(A)** Identification of the power of each explanatory phenotypic variable to the genotype discrimination. GLM, Generalized Linear Model; RF, Random Forest. The explanatory variables selected were the ones found to contribute more than a 30% to the genotype discrimination. **(B)** Identification of the power of the explanatory phenotypic variables contributing to more than 30% to the genotype discrimination, analyzed to see for different responses based on sex and using the two different statistical GLM and RF classifiers. **(C)** 3D-PCA plots showing the individual animals clustering in 3D space based on PCA analyses performed with all the phenotypic variables and colors based on the genotype and sex. Left panel shows individuals of both sexes, the middle plots female data, and the right plot male data. **(D,E)** The qualitative variable discrimination component maps show the distribution in 2D space of the qualitative variables coordinates calculated based on PCA analysis performed with the multifactor analysis of mixed data (MFA). In panel **(D)**, the variables female, male, wt, het, and homo are represented. In panel **(E)**, the variables genotype, sex, tail flick, acetone, cold plate, and von Frey are shown.

We analyzed how much each behavioral variable could discriminate between the three genotypes (wt, heterozygous noted as het, and homozygous noted as homo) and between the two sexes. To perform the Gdaphen analysis, we considered 18 variables in total, genotype, sex, and 16 behavioral variables. Among these 18 variables, 6 were detected as the most relevant (> 30%) to discriminate among the 3 genotypes. These variables were sex, von Frey threshold, cold plate paw lifts, number of paw reactions, and paw reaction duration in the acetone test and tail flick latency. For discriminating between the three genotypes, the GLM classifier identified the von Frey threshold, cold plate paw lifts, and the number of paw reactions in the acetone test as the major variables discriminating between the three genotypes following a linear gene dosage effect. Instead, the RF classifier found von Frey threshold and the number and duration of paw reactions in the acetone test as the most discriminating parameters (Figure 5A). Interestingly sex dimorphism was shown to be contributing to the homozygous genotype discrimination by RF. For sex discrimination, GLM and RF (Figure 5B) identified paw reaction duration in the acetone test as the most important variable, followed by Hargreaves threshold. In addition, GLM identified von Frey threshold and cold plate paw lifts as important discriminant variables and RF identified tail pressure. The 3D-PCA plots (Figure 5C) show the clustering of individual animals in the 3D space based on PCA analyses performed with all the phenotypic variables and colored them based on the genotype and sex. A clear genotype clustering can be seen in the left panel showing the three genotypes and two-sex individuals, in the middle plot for female data only, and in the right plot for male data. As can be seen in Figure 5D, based on the qualitative variable discrimination 2D component map, the genotype effect was stronger than the sex effect for the correct clustering of individuals. Furthermore, the PCA component 1 was mostly explaining the variability accounting to the wt and homozygous genotypes and the component 3 denotes the heterozygous genotype. Dimension 2 was strongly explaining the sex effect in females and males. Finally, all three PCA components explained the variability accounting to the genotype and von Frey variables, components 2 and 1 for sex, component 1 for behavior in the acetone and tail flick tests, and components 2 and 3 for cold plate (Figure 5E).

### The *Scn9a*^*R*185*H*^ Mice Show Spontaneous Ongoing Pain

In order to assess the presence of ongoing pain in *Scn9a*^*R*185*H*^ mice, we employed a CPP test. This assay is based on the positive reinforcement provided by the non-rewarding analgesic, clonidine. Animals with ongoing pain prefer the compartment where they received clonidine as compared to the compartment where they got the saline control solution.

**FIGURE 6:**
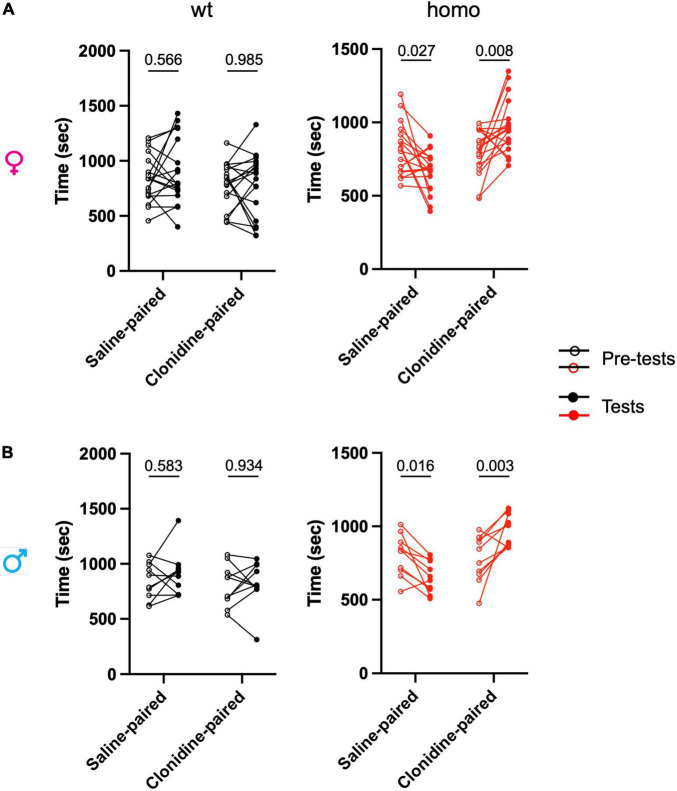
*Scn9a*^*R*185*H*^ mutant mice show spontaneous ongoing pain. Ongoing pain was assessed in a conditioned place preference test. After pre-test/habituation for chamber preference on days 1 to 3, conditioning was made on day 4 by intrathecal treatment with either saline solution in the morning or clonidine solution in the afternoon with a restriction in one chamber. On day 5, the animals were free to explore the chambers and the time spent in each chamber was recorded for 30 min. **(A)** Females. **(B)** Males. The time spent in each chamber before conditioning (pre-test) and after conditioning with clonidine or saline (test) are shown. Homozygous mice of both sexes spent more time in clonidine-paired chamber while wt mice spent on average equal time in saline-paired and clonidine-paired chambers. Dots represent individual data points. females: wt, *n* = 18; Homo, *n* = 18; males: wt, *n* = 10; Homo, *n* = 10. Two-way ANOVA for treatment and trial (pretest and test) on each genotype and sex group, Sidak’s multiple comparison test. *P* = values for comparing pre-test and test values are shown. Refer to Supplementary Table 12 for statistics.

The three-way ANOVA showed an interaction between the genotype, solutionpaired chamber (saline vs clonidine), and trial (pre-test vs. test) in both males and females (Figure 6 and Supplementary Table 12). Additional two-way ANOVA of time spent in the clonidine-paired chamber compared to the saline-paired chamber in pre-test vs. test trials showed a strong treatment effect (clonidine vs. saline) in homozygous animals and not in wt animals, of both sexes (Figure 6 and Supplementary Table 12).

The wt mice showed no preference for either compartment, while homozygous mice of both sexes preferred the clonidine-paired chamber. These results indicate the presence of ongoing pain in both female and male homozygous mutants.

### Influence of Sex on Pain Behavioral Responses in Wild-Type Animals

For all pain behavioral tests, the effects of both mutation and sex have been analyzed by two-way ANOVAs as described above. In order to better apprehend the influence of sex on these pain responses, the behavior of wt females and males was compared (refer to Supplementary Figure 7 and Supplementary Table 13). This shows that, concerning the heat modality, wt females tend to be more sensitive than wt males on the 56°C hot plate for the first response latency and coping reactions (Supplementary Figures 7A,B), and equally sensitive for all other hot plate parameters and in the tail flick and Hargreaves models (Supplementary Figures 7A–D). The wt females and males showed a different temperature preference profile in the thermal gradient ring (Supplementary Figure 7E). For sensitivity to cool temperatures, wt females showed longer durations of paw reactions as compared to males in the acetone test, and no sex influence was detected in the cold plate assay (Supplementary Figures 7F,G). In response to non-noxious mechanical stimulation as assessed by the von Frey test, wt females were shown to be more sensitive than wt males in the INCI institute (Supplementary Figure 7H). Regardless of this sex difference, homozygous mutant animals of both sexes were found more sensitive than their wt counterparts, indicating that the *Scn9a*^*R*185*H*^ mutation has a strong impact on sensitivity to touch. No sex difference in the sensitivity to pressure was detected in wt animals (Supplementary Figure 7I).

When spontaneous pain was evaluated in the CPP, the two-way ANOVA showed no clonidine-paired chamber preference in wt animals (Supplementary Figure 7J), indicating the absence of spontaneous pain. Also, no sex bias for the time spent in the clonidine-paired chamber as compared to the saline-paired chamber was found. The spontaneous pain evidenced in mutants of both sexes thus suggests that the *Scn9a*^*R*185*H*^ mutation induces pain in both males and females which is not paired to a sex susceptibility in wt animals.

### The *Scn9a*^*R*185*H*^ Mice Do Not Show Anxio-Depressive Behavioral Consequences of Chronic Pain

The homozygous *Scn9a*^*R*185*H*^ mutants were evaluated for the anxio-depressive consequences of chronic pain in two classical paradigms, the light-dark box for anxiety-like behavior and the forced-swim test for despair-like behavior. The mutants behaved comparably to their wt counterparts when analyzed in the light-dark box (Supplementary Figure 6B and Supplementary Table 14). Similarly, mutant mice spent similar immobility time as their wt controls in the forced-swim test (Supplementary Figure 6C and Supplementary Table 14). These results suggest that the *Scn9a*^*R*185*H*^ mutation alters sensitivity to thermal and mechanical stimuli and produces spontaneous ongoing pain but does not induce anxiodepressive-like consequences along the tests used in this study.

## Discussion

In this study, we successfully established, by using the CRISPR-Cas9 technique, the *Scn9a*^*R*185*H*^ mutant mouse line as a model for the human *SCN9A*^*R*185*H*^ mutation found in patients with SFN having chronic pain. The wt control mice highly expressed *Scn9a* mRNA in DRG and less in spinal cord, in agreement with previous findings. No alteration of *Scn9a* mRNA or protein expression level was detected in *Scn9a*^*R*185*H*^ mutant mice, suggesting that the behavioral changes in mutants may not be attributed to alterations in Nav1.7 expression. Our results indicate that *Scn9a*^*R*185*H*^ mice show a pain phenotype in a mutation-dosage dependent manner, suggesting that the *Scn9a*^*R*185*H*^ mutation identified in patients with SFN having chronic pain contributes for their pain symptoms. This is confirmed in a different species, the *SCN9A*^*R*185*H*^ mutation, as a pathogenic mutation.

Three patients with *SCN9A*^*R*185*H*^ having chronic pain had decreased intraepidermal nerve fibers density (IENFD). Although IENFD still remains to be investigated in *Scn9a*^*R*185*H*^ mice, the impact of the mutation was investigated on both DRGs and sciatic nerves. Size distribution of DRG neurons was similar in the three genotypes, indicating that the mutation did not induce a selective loss of small size DRG neurons. In addition, the density of PGP9.5 and Nav1.7 labeling was comparable in sciatic nerves of all three genotypes, suggesting that the mutation did not lead to a decrease in sciatic fibers. This is the first description of sensory neurons in the *Scn9a*^*R*185*H*^ GOF mutant mice, while DRG and sciatic nerves cannot be investigated in patients and healthy individuals. Taken globally, the R185H mutation produced neuron hyperexcitability when transfected *in vitro* into rat DRG neurons (Han et al., 2012) and, when introduced in mice, leading to no obvious sensory nerve damage, an increased sensitivity in several pain tests and ongoing spontaneous pain. On the other hand, another mutation found in patients with chronic pain having reduced IENFD, the *SCN9A*^*I*228*M*^ mutation, also led to DRG neuron hyperexcitability *in vitro* but to no neuropathy and no pain behavior phenotype when introduced into mice (Chen et al., 2021). The *SCN10A*^*G*1662*S*^ mutation found in pain patients with normal IENFD did produce hyperexcitability when transfected into DRG neurons (Han et al., 2014; Xiao et al., 2019) as well as normal IENFD and increased pain sensitivity in *Scn10a*^*G*1663*S*^ mice (Chidiac et al., 2021). Therefore, the comparison of these data on the three mutations indicates no strict correlation between *in vitro* hyperexcitability of DRG neurons, neuropathy, and pain phenotype of mutant mice. A difference between patients with chronic pain and the *in vitro* and *in vivo* animal models described above resides in different time courses, in terms of days, weeks, and years, which may possibly lead to different adaptative mechanisms. The different genetic backgrounds in these species may also differentially impact on the mutation effects. Of note, the study by Bianca de Greef et al. (2018) showed that, out of 921 patients with possible SFN diagnosis, 614 had normal IENFD and abnormal temperature sensitivity threshold, demonstrating that abnormal IENFD is a secondary criterion for painful SFN in these patients, and the consequences of the *Scn9a*^*R*185*H*^ GOF mutation on the spinal and brain pain pathways may be further investigated. More generally, the link between Nav channels GOF mutations, sensory neuron activity, neuropathy, and pain feeling will be further investigated when more tools become available.

### Heat Pain in *Scn9a*^*R*185*H*^ Mice

Patients with SFN s harboring the *SCN9A*^*R*185*H*^ mutation complained of pain and paresthesias with burning feet and abnormal warm and cold pain sensation as evaluated by quantitative sensory testing (Faber et al., 2012). In *Scn9a*^*R*185*H*^ mutant mice, pain behavior phenotype was observed in the hot plate and tail flick tests. This suggests that we successfully transferred the heat pain symptoms with *SCN9A*^*R*185*H*^ mutation in our mutant line. The heat-sensitivity phenotype was found with a gene dosage effect, strengthening the conclusion on the impact of the mutation. Mutant females were found more sensitive than male littermates and the sex effect will be further discussed below. In addition, temperature preference as assessed between 15 and 40°C was not affected by the mutation, indicating that this mutation does not change the temperature sensing profile at non-noxious temperatures. As compared, deleting Nav1.7 in C-low threshold mechanoreceptors (C-LTMs) led to a shift in preferred temperature from 28.8 to 24°C (Middleton et al., 2021). Taken together, we showed hypersensitivity to noxious heat in *Scn9a*^*R*185*H*^ mice while former studies had demonstrated an analgesic phenotype in *Scn9a* KO mouse lines (Xue et al., 2021). Taken globally, this suggests that the *Scn9a*^*R*185*H*^ mutation induces hypersensitivity to heat.

### Cold Pain in *Scn9a*^*R*185*H*^ Mice

In the *Scn9a*^*R*185*H*^ line, males and not females showed more reactions to cooling in the acetone test. Interestingly, the “acetone duration paw reaction” is one of the most discriminating variables linked to the genotype. The increased sensitivity to cooling as assessed in the acetone test is in agreement with previous findings which showed that Nav1.8 and Nav1.9 channels in sensory neurons display unusual biophysical adaptations to operate during extreme cold while Nav1.7 channels are necessary for cooling but not extreme cold sensation as well as for surgery-induced neuropathic cool allodynia (Minett et al., 2012, 2014a,b; Pereira et al., 2018; Shields et al., 2018). Also, the genetic mouse cKO models have globally shown that *Scn9a* is involved in acetone cool pain rather than cold plate cold behavioral responses (Xue et al., 2021). The findings of altered cool responses in *Scn9a*^*R*185*H*^ mice are consistent with the description of the two patients with *R185H* presenting cold impaired modalities in quantitative sensory tests (Faber et al., 2012).

### Mechanical Hypersensitivity in *Scn9a*^*R*185*H*^ Mice

Both *Scn9a*^*R*185*H*^ males and females showed a reduced mechanical threshold in the von Frey test with a mutation-dosage effect. They had normal noxious thresholds in tail pressure, indicating that *Scn9a*^*R*185*H*^ mutation may cause mechanical allodynia rather than hyperalgesia. Regarding genetic rodent models, rats with *Scn9a* loss-of-function and global *Scn9a* KO mice were reported to display reduced sensitivity to von Frey and pinprick tests as well as to the Randall-Selitto or tail clip tests (Gingras et al., 2014; Grubinska et al., 2019; Chen L. et al., 2020; Xue et al., 2021). Altogether, our current findings and the cited previous results suggest that lack of *Scn9a* leads to less responses to both innocuous and noxious mechanical stimuli, whereas the *Scn9a*^*R*185*H*^ mutation induces mechanical-touch allodynia rather than hyperalgesia to noxious pressure. These results on *Scn9a*^*R*185*H*^ mice coincide with *R185H* patients’ complaint of sheet intolerance.

### Spontaneous Ongoing Pain in *Scn9a*^*R*185*H*^ Mice

Measures of reflexive behaviors, such as withdrawals from noxious stimuli have been used for decades to examine pain behaviors. Here, we successfully validated the *Scn9a*^*R*185*H*^ mutant mouse model and found a pain phenotype by examining reflexive pain behaviors. However, pain is a multidimensional experience of sensory-discriminative, cognitive, and affective processes. More recently, preference for the compartment paired with analgesia as measured by the CPP, or avoidance for evoked stimuli as recorded by the conditioned place avoidance test, have been used to assess spontaneous pain behavior, i.e., the presence of ongoing pain in experimentally induced inflammatory and neuropathic pain conditions (King et al., 2009; Barrot, 2012; Barthas et al., 2015) The *Scn9a*^*R*185*H*^ mutant mice show a preference for the analgesia-paired chamber in the CPP test, indicating ongoing pain. These results together with our findings on the other behaviors indicate that *Scn9a*^*R*185*H*^ mutant mice do not only have altered nociceptive levels but also experience spontaneous pain, that corresponds more closely to the verbal reports of patients.

### Sex Influence on Behaviors of *Scn9a*^*R*185*H*^ Mutants

This first evaluation of the behavioral consequences of the *Scn9a*^*R*185*H*^ mutation showed specific sex differences. Indeed, *Scn9a*^*R*185*H*^ females show more pain-like behaviors to heat (jump latency, coping reactions on hot plate and tail flick) while mutant males display more cool-related reactions (acetone). And notably, the increased sensitivity to touch and spontaneous pain were not sexually dimorphic. The study by Faber et al. (2012) comprising both male and female patients with *SCN9A*^*R*185*H*^ showed that the mutation was associated with pain in individuals of both sexes. In fact, this analysis was done in rare patients, one man with complaints and pain abnormalities similar to his brother and grand-father, and one woman whose father had similar complaints; thus, a quantitative conclusion cannot be drawn. Although these were rare patients and a quantitative conclusion cannot be drawn, generally, a majority of patients with chronic pain who visited the clinics are found to be women.

The current available evidence indicates hormonal, genetic, neuroimmune, cognitive, and social factors for the establishment and maintenance of chronic pain. Concerning rodents, sex differences or no sex differences in pain were reported in some strains but not in others (Mogil, 2020; Sadler et al., 2022). Interestingly, sex effects in *Scn9a*^*R*185*H*^ mutant mice may be explained by a differential effect of the mutation on Nav1.7 localization at the plasma membrane. Indeed, recently Nav1.7 membrane localization was shown to be regulated by the SUMOylation of the collapsin response mediator protein 2 (CRMP2) in female but not in the male sensory neurons (Moutal et al., 2020). This sex-specific mechanism may be tested on *Scn9a*^*R*185*H*^ animals on further investigations.

### Perspectives

The present new genetic model, together with previous Nav1.7/*Scn9a* models and investigations have shown that Nav1.7 plays important roles in nociception and chronic pain. Although there are differences between rodents and humans, the present genetic mouse model may help to understand the impact of *Scn9a*^*R*185*H*^ mutation. Pain is a multifaceted and diverse experience that can be categorized into several types and modalities depending on the presentation and triggering stimulus of the pain event. The analyzed behaviors could provide a first phenotyping of the *Scn9a*^*R*185*H*^ mutant animals, and the study may be extended to other clinically relevant behavioral assays to capture the full spectrum of pain in these mice and open new visions for pain therapies based on Nav1.7. In addition, functional magnetic resonance imaging (fMRI) could be used next to study the neural activity underlying *Scn9a*^*R*185*H*^ mutation-induced pain. Recently, several emerging imaging methods have been developed (Geha et al., 2016; Chen X. et al., 2020; Markicevic et al., 2021) which may be applied to investigate the consequences of *Scn9a*^*R*185*H*^ mutation in the brain of the mouse.

In addition, *Scn9a*^*R*185*H*^ mice may constitute a tool to further define mechanisms linked to Nav1.7 interaction with other proteins. As mentioned above, Nav1.7 interacts with SUMOylated CRMP2 to regulate its membrane location (Moutal et al., 2020). The impact of *Scn9a*^*R*185*H*^ mutation on cell location may be explored further to elucidate the increased pain observed in the mutant animals. Also, targeted *in situ* repression of Nav1.7 in primary afferent mice led to long-lasting analgesia (Moreno et al., 2021) and the pain insensitivity found in *Scn9a* null mice was shown to be associated to elevated opioid receptors activity (Pereira et al., 2018; MacDonald et al., 2021). This revealed the requirement of both Nav1.7 absence and elevated opioid activity for a full analgesia. Also, the lack of success in developing Na_*V*_ channel blockers was reported to be due to their lack of specificity to Nav1.7 but previous findings on *Scn9a* null mice suggested that this may also be due to their lack of effect on the endogenous opioids. Thus, profiling of the endogenous opioid analgesic system could be next addressed in *Scn9a*^*R*185*H*^ mice. Another component of neuropathic pain lies in neuroinflammation (Sommer et al., 2018; Sisignano et al., 2022). The impact of *Scn9a*^*R*185*H*^ mutation on neuroinflammation or on a broader scale may be further explored by omics approaches.

Also, the limited results of clinical trials with Na_*V*_ channel blockers may reside in the differences between human and rodent DRG neurons. Hence, the development of iPSC-derived human sensory neurons, including those from *SCN9A*^*R*185*H*^ patients may provide a new preclinical platform for the *in vitro* design of next pain therapies (Alsaloum and Waxman, 2022). Altogether, the combination of rodent genetic models and other molecular, genetic, cellular, and pharmacological approaches will allow to identify additional components of chronic pain and to design new therapeutic strategies (Calvo et al., 2019; Kingwell, 2021).

## Data Availability Statement

The original contributions presented in the study are included in the article/Supplementary Material, further inquiries can be directed to the corresponding authors.

## Ethics Statement

The animal study was reviewed and approved by Com’Eth, «Comité d’Ethique pour l’Expérimentation Animale IGBMC-ICS, and CREMEAS».

## Author Contributions

YX, MK, MM, CC, RL, M-CB, MB, YH, and CG-R contributed to the conception and design of the study. YX, MK, and RL performed experiments. YX, MK, MM, RL, M-CB, MB, and CG-R analyzed data. All authors contributed to the writing of the manuscript and approved the submitted version.

## Conflict of Interest

The authors declare that the research was conducted in the absence of any commercial or financial relationships that could be construed as a potential conflict of interest.

## Publisher’s Note

All claims expressed in this article are solely those of the authors and do not necessarily represent those of their affiliated organizations, or those of the publisher, the editors and the reviewers. Any product that may be evaluated in this article, or claim that may be made by its manufacturer, is not guaranteed or endorsed by the publisher.

## Footnotes

1
http://crispor.tefor.net/crispor.py

2
http://www.ensembl.org/index.html
