# Supplementary material for: The Human SCN9AR185H Point Mutation Induces Pain Hypersensitivity and Spontaneous Pain in Mice
Source: Front Mol Neurosci. 2022 Jun 13;15:913990. doi: 10.3389/fnmol.2022.913990 (PMC9234669; doi:10.3389/fnmol.2022.913990)
Supplement: Supplementary file 1 [file Data_Sheet_1.PDF]

The human *SCN9A*^{R185H} point mutation induces pain hypersensitivity and spontaneous pain in mice

Yaping Xue, Mélanie Kremer, Maria del Mar Muniz Moreno, Celeste Chidiac, Romain Lorentz, Marie-Christine Birling, Michel Barrot, Yann Herault and Claire Gaveriaux-Ruff

Supplementary Material

```
Scn9a_exon5_WT_ref     GTACACTTTTACTGGGATATATACTTTTGAATCACTCATAAAAATCCTTGCAAGAGGCTTTTGCGTGGGCGAATTCACCTTCCTCCG-TGACCCTTGGAACTGGCTGGACTTTGTTGTCATTGTTTTTGC

21#_ins_1nt            GTACACTTTTACTGGGATATATACTTTTGAATCACTCATAAAAATCCTTGCAAGAGGCTTTTGCGTGGGCGAATTCACCTTCCTCCGTTGACCCTTGGAACTGGCTGGACTTTGTTGTCATTGTTTTTGC
21#_del_21nt           GTACACTTTTACTGGGATATATACTTTTGAATCACTCATAAAAATCCTTGCAAGAGGCTTTTGCGTGGGCGAATTCACCTTCCT----------------------GGACTTTGTTGTCATTGTTTTTGC

26#_ins_1nt            GTACACTTTTACTGGGATATATACTTTTGAATCACTCATAAAAATCCTTGCAAGAGGCTTTTGCGTGGGCGAATTCACCTTCCTCCGT------TGACCCTTGGAACTGGCTGGACTTTGTTGTCATTGTTTTTGC
26#_del_1nt_ins_7nt    GTACACTTTTACTGGGATATATACTTTTGAATCACTCATAAAAATCCTTGCAAGAGGCTTTTGCGTGGGCGAATTCACCTTCCTCC-TTCTTCATGACCCTTGGAACTGGCTGGACTTTGTTGTCATTGTTTTTGC

28#_PM                 GTACACTTTTACTGGGATATATACTTTTGAATCACTCATAAAAATCCTTGCAAGAGGCTTTTGCGTGGGCGAATTCACCTTTCTTCA--------TGACCCTTGGAACTGGCTGGACTTTGTTGTCATTGTTTTGC
28#_del_1nt_ins_7nt    GTACACTTTTACTGGGATATATACTTTTGAATCACTCATAAAAATCCTTGCAAGAGGCTTTTGCGTGGGCGAATTCACCTTTCTTC-TTTCCCCATGACCCTTGGAACTGGCTGGACTTTGTTGTCATTGTTTTGC

29#_WT                 GTACACTTTTACTGGGATATATACTTTTGAATCACTCATAAAAATCCTTGCAAGAGGCTTTTGCGTGGGCGAATTCACCTTCCTCCG-TGACCCTTGGAACTGGCTGGACTTTGTTGTCATTGTTTTTGC
29#_ins_1nt_PM         GTACACTTTTACTGGGATATATACTTTTGAATCACTCATAAAAATCCTTGCAAGAGGCTTTTGCGTGGGCGAATTCACCTTTCTTCATTGACCCTTGGAACTGGCTGGACTTTGTTGTCATTGTTTTTGC

31#_PM_hom             GTACACTTTTACTGGGATATATACTTTTGAATCACTCATAAAAATCCTTGCAAGAGGCTTTTGCGTGGGCGAATTCACCTTTCTTCATGACCCTTGGAACTGGCTGGACTTTGTTGTCATTGTTTTTGC

34#_WT                 GTACACTTTTACTGGGATATATACTTTTGAATCACTCATAAAAATCCTTGCAAGAGGCTTTTGCGTGGGCGAATTCACCTTCCTCCG--TGACCCTTGGAACTGGCTGGACTTTGTTGTCATTGTTTTTGC
34#_ins_2nt_PM         GTACACTTTTACTGGGATATATACTTTTGAATCACTCATAAAAATCCTTGCAAGAGGCTTTTGCGTGGGCGAATTCACCTTTCTTCAAATGACCCTTGGAACTGGCTGGACTTTGTTGTCATTGTTTTTGC

36#_WT                 GTACACTTTTACTGGGATATATACTTTTGAATCACTCATAAAAATCCTTGCAAGAGGCTTTTGCGTGGGCGAATTCACCTTCCTCCG-------TGACCCTTGGAACTGGCTGGACTTTGTTGTCATTG
36#_del_2nt            GTACACTTTTACTGGGATATATACTTTTGAATCACTCATAAAAATCCTTGCAAGAGGCTTTTGCGTGGGCGAATTCACCTTCCTT---------TGACCCTTGGAACTGGCTGGACTTTGTTGTCATTG
36#_del_1nt            GTACACTTTTACTGGGATATATACTTTTGAATCACTCATAAAAATCCTTGCAAGAGGCTTTTGCGTGGGCGAATTCACCTTCCTCCG--------GACCCTTGGAACTGGCTGGACTTTGTTGTCATTG
36#_ins_7nt            GTACACTTTTACTGGGATATATACTTTTGAATCACTCATAAAAATCCTTGCAAGAGGCTTTTGCGTGGGCGAATTCACCTTCCTCCGTCCGTGATGACCCTTGGAACTGGCTGGACTTTGTTGTCATTG

37#_WT                 GTACACTTTTACTGGGATATATACTTTTGAATCACTCATAAAAATCCTTGCAAGAGGCTTTTGCGTGGGCGAATTCACCTTCCTCCG-TGACCCTTGGAACTGGCTGGACTTTGTTGTCATTGTTTTTGC
37#_ins_1nt_PM         GTACACTTTTACTGGGATATATACTTTTGAATCACTCATAAAAATCCTTGCAAGAGGCTTTTGCGTGGGCGAATTCACCTTTCTTCATTGACCCTTGGAACTGGCTGGACTTTGTTGTCATTGTTTTTGC

38#_WT                 GTACACTTTTACTGGGATATATACTTTTGAATCACTCATAAAAATCCTTGCAAGAGGCTTTTGCGTGGGCGAATTCACCTTCCTCCG-TGACCCTTGGAACTGGCTGGACTTTGTTGTCATTGTTTTTGC
38#_ins_1nt_PM         GTACACTTTTACTGGGATATATACTTTTGAATCACTCATAAAAATCCTTGCAAGAGGCTTTTGCGTGGGCGAATTCACCTTTCTTCATTGACCCTTGGAACTGGCTGGACTTTGTTGTCATTGTTTTTGC

41#_del_1nt_PM         GTACACTTTTACTGGGATATATACTTTTGAATCACTCATAAAAATCCTTGCAAGAGGCTTTTGCGTGGGCGAATTCACCTTTCTTCATGACCCTTG-AACTGGCTGGACTTTGTTGTCATTGTTTTTGC
41#_ins_3nt_del_6nt    GTACACTTTTACTGGGATATATACTTTTGAATCACTCATAAAAATCCTTGCAAGAGGCTTTTGCGTGGGCGAATTCACCTTCTT------CCCTTGGAACTGGCTGGACTTTGTTGTCATTGTTTTTGC

42#_WT                 GTACACTTTTACTGGGATATATACTTTTGAATCACTCATAAAAATCCTTGCAAGAGGCTTTTGCGTGGGCGAATTCACCTTCCTCCGTGACCCTTGGAACTGGCTGGACTTTGTTGTCATTGTTTTTGC
42#_del_6nt            GTACACTTTTACTGGGATATATACTTTTGAATCACTCATAAAAATCCTTGCAAGAGGCTTTTGCGTGGGCGAATTCACCTTTCT------CCCTTGGAACTGGCTGGACTTTGTTGTCATTGTTTTTGC
42#_PM                 GTACACTTTTACTGGGATATATACTTTTGAATCACTCATAAAAATCCTTGCAAGAGGCTTTTGCGTGGGCGAATTCACCTTTCTTCATGACCCTTGGAACTGGCTGGACTTTGTTGTCATTGTTTTTGC

43#_WT                 GTACACTTTTACTGGGATATATACTTTTGAATCACTCATAAAAATCCTTGCAAGAGGCTTTTGCGTGGGCGAATTCACCTTCCTCCG-TGACCCTTGGAACTGGCTGGACTTTGTTGTCATTGTTTTTGC
43#_del_6nt            GTACACTTTTACTGGGATATATACTTTTGAATCACTCATAAAAATCCTTGCAAGAGGCTTTTGCGTGGGCGAATTCACCTTTCT-------CCCTTGGAACTGGCTGGACTTTGTTGTCATTGTTTTTGC
43#_ins_1nt_PM         GTACACTTTTACTGGGATATATACTTTTGAATCACTCATAAAAATCCTTGCAAGAGGCTTTTGCGTGGGCGAATTCACCTTTCTTCATTGACCCTTGGAACTGGCTGGACTTTGTTGTCATTGTTTTTGC

45#_PM                 GTACACTTTTACTGGGATATATACTTTTGAATCACTCATAAAAATCCTTGCAAGAGGCTTTTGCGTGGGCGAATTCACCTTTCTTCA-------TGACCCTTGGAACTGGCTGGACTTTGTTGTCATTGTTTTTGC
45#_del_1nt_ins_7nt    GTACACTTTTACTGGGATATATACTTTTGAATCACTCATAAAAATCCTTGCAAGAGGCTTTTGCGTGGGCGAATTCACCTTCCTCC-TTCTTCATGACCCTTGGAACTGGCTGGACTTTGTTGTCATTGTTTTTGC

46#_PM                 GTACACTTTTACTGGGATATATACTTTTGAATCACTCATAAAAATCCTTGCAAGAGGCTTTTGCGTGGGCGAATTCACCTTTCTTCATGACCCTTGGAACTGGCTGGACTTTGTTGTCATTGTTTTTGC
46#_del_14nt           GTACACTTTTACTGGGATATATACTTTTGAATCACTCATAAAAATCCTTGCAAGAGGCTTTT--------------ACCTTTCTTCATGACCCTTGGAACTGGCTGGACTTTGTTGTCATTGTTTTTGC
46#_del_2nt            GTACACTTTTACTGGGATATATACTTTTGAATCACTCATAAAAATCCTTGCAAGAGGCTTTTGCGTGGGCGAATTCACCTTCCTC--TGACCCTTGGAACTGGCTGGACTTTGTTGTCATTGTTTTTGC

49#_WT                 GTACACTTTTACTGGGATATATACTTTTGAATCACTCATAAAAATCCTTGCAAGAGGCTTTTGCGTGGGCGAATTCACCTTCCTCCG-TGACCCTTGGAACTGGCTGGACTTTGTTGTCATTGTTTTTG
49#_ins_1nt_PM         GTACACTTTTACTGGGATATATACTTTTGAATCACTCATAAAAATCCTTGCAAGAGGCTTTTGCGTGGGCGAATTCACCTTTCTTCATTGACCCTTGGAACTGGCTGGACTTTGTTGTCATTGTTTTTG

51#_WT                 GTACACTTTTACTGGGATATATACTTTTGAATCACTCATAAAAATCCTTGCAAGAGGCTTTTGCGTGGGCGAATTCACCTTCCTCCG----TGACCCTTGGAACTGGCTGGACTTTGTTGTCATTGTTTTTGC
51#_ins_4nt_del_6nt    GTACACTTTTACTGGGATATATACTTTTGAATCACTCATAAAAATCCTTGCAAGAGGCTTTTGCGTGGGCGAATTCACCTTCCTCCATCTC------TTGGAACTGGCTGGACTTTGTTGTCATTGTTTTTGC
51#_ins_1nt            GTACACTTTTACTGGGATATATACTTTTGAATCACTCATAAAAATCCTTGCAAGAGGCTTTTGCGTGGGCGAATTCACCTTTCTTCGG---TGACCCTTGGAACTGGCTGGACTTTGTTGTCATTGTTTTTGC

52#_WT                 GTACACTTTTACTGGGATATATACTTTTGAATCACTCATAAAAATCCTTGCAAGAGGCTTTTGCGTGGGCGAATTCACCTTCCTCC-G--TGACCCTTGGAACTGGCTGGACTTTGTTGTCATTGTTTTGC
52#_ins_2nt            GTACACTTTTACTGGGATATATACTTTTGAATCACTCATAAAAATCCTTGCAAGAGGCTTTTGCGTGGGCGAATTCACCTTTCTTC-ATGTGACCCTTGGAACTGGCTGGACTTTGTTGTCATTGTTTTGC
52#_ins_1nt            GTACACTTTTACTGGGATATATACTTTTGAATCACTCATAAAAATCCTTGCAAGAGGCTTTTGCGTGGGCGAATTCACCTTCCTCCCG--TGACCCTTGGAACTGGCTGGACTTTGTTGTCATTGTTTTGC

55#_PM_homo            GTACACTTTTACTGGGATATATACTTTTGAATCACTCATAAAAATCCTTGCAAGAGGCTTTTGCGTGGGCGAATTCACCTTTCTTCATGACCCTTGGAACTGGCTGGACTTTGTTGTCATTGTTTTTGC

55#_PM                 GTACACTTTTACTGGGATATATACTTTTGAATCACTCATAAAAATCCTTGCAAGAGGCTTTTGCGTGGGCGAATTCACCTTTCTCCATGACCCTTGGAACTGGCTGGACTTTGTTGTCATTGTTTTTGC
55#_del_6nt            GTACACTTTTACTGGGATATATACTTTTGAATCACTCATAAAAATCCTTGCAAGAGGCTTTTGCGTGGGCGAATTCACCTTTCT------CCCTTGGAACTGGCTGGACTTTGTTGTCATTGTTTTTGC

57#_PM                 GTACACTTTTACTGGGATATATACTTTTGAATCACTCATAAAAATCCTTGCAAGAGGCTTTTGCGTGGGCGAATTCACCTTTCTTCATGACCCTTGGAACTGGCTGGACTTTGTTGTCATTGTTTTTGC
57#_del_7nt            GTACACTTTTACTGGGATATATACTTTTGAATCACTCATAAAAATCCTTGCAAGAGGCTTTTGCGTGGGCGAATTCACCTTC-------ACCCTTGGAACTGGCTGGACTTTGTTGTCATTGTTTTTGC

58#_WT                 GTACACTTTTACTGGGATATATACTTTTGAATCACTCATAAAAATCCTTGCAAGAGGCTTTTGCGTGGGCGAATTCACCTTCCTCCG-TGACCCTTGGAACTGGCTGGACTTTGTTGTCATTGTTTTTGC
58#_ins_1nt            GTACACTTTTACTGGGATATATACTTTTGAATCACTCATAAAAATCCTTGCAAGAGGCTTTTGCGTGGGCGAATTCACCTTTCTTCATTGACCCTTGGAACTGGCTGGACTTTGTTGTCATTGTTTTTGC
```

Supplementary Figure 1. Genomic DNA analysis shows *Scn9a* gene editing in F0 mice. Sequence analysis by Sanger sequencing confirms on-target mutagenesis with a wide range of alleles containing Homology Directed Repair events with point mutation or Non-Homologous End Joining events with indel mutations including different insertions and deletions. Sequencing results for founders until #58 are shown.

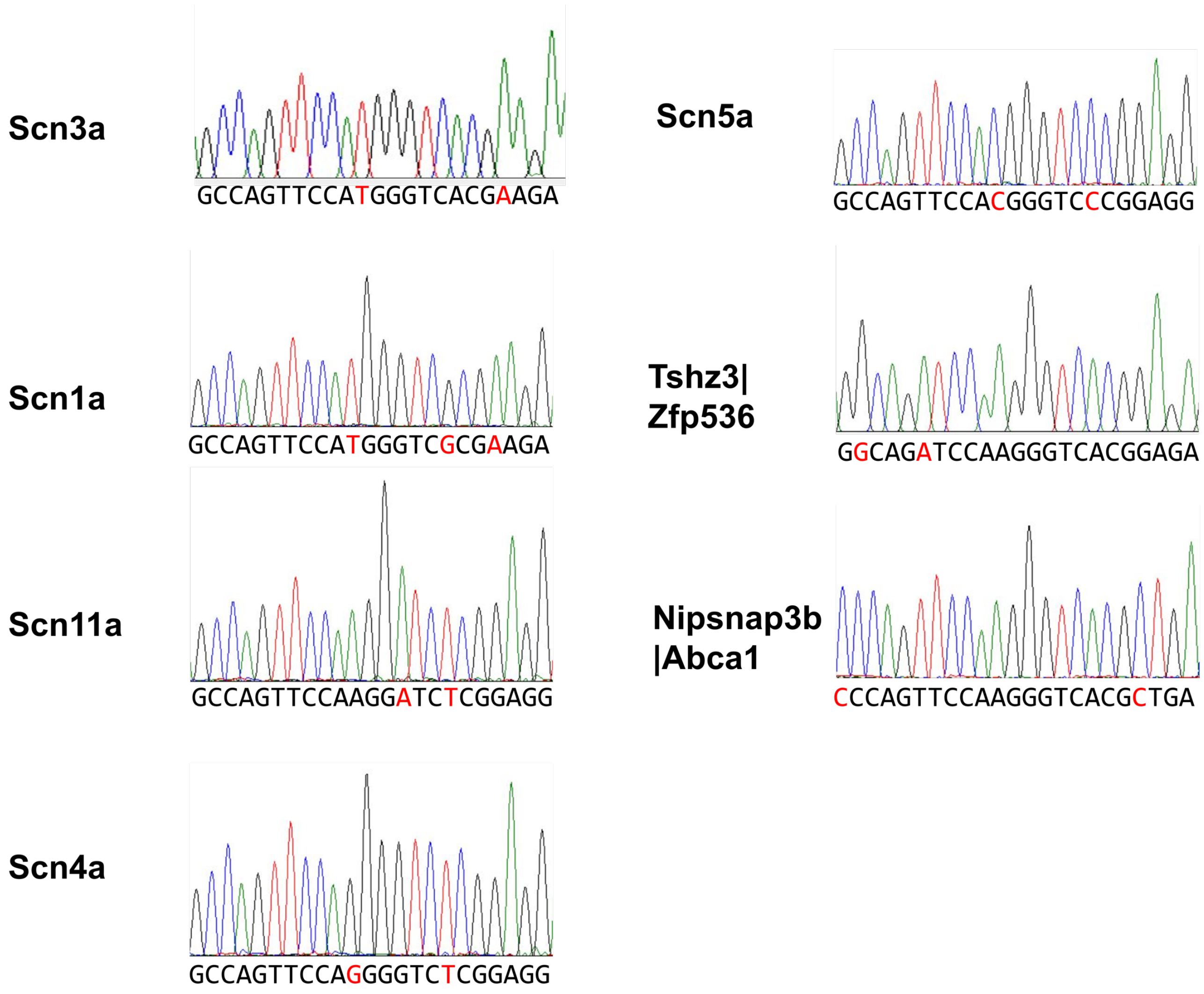

Supplementary Figure 2. The analysis of off-targets in the F1 mouse #53-11 used as the founder to establish the *Scn9a*^{R185H} mouse line. Seven important potential off target genes, including gene *Scn3a, Scn1a, Scn11a, Scn4a, Scn5a* and two locations between two genes (Tshz3/Zfp536 and Nipsnap3b/Abca1) have been checked in this F1 founder. No off-target was detected.

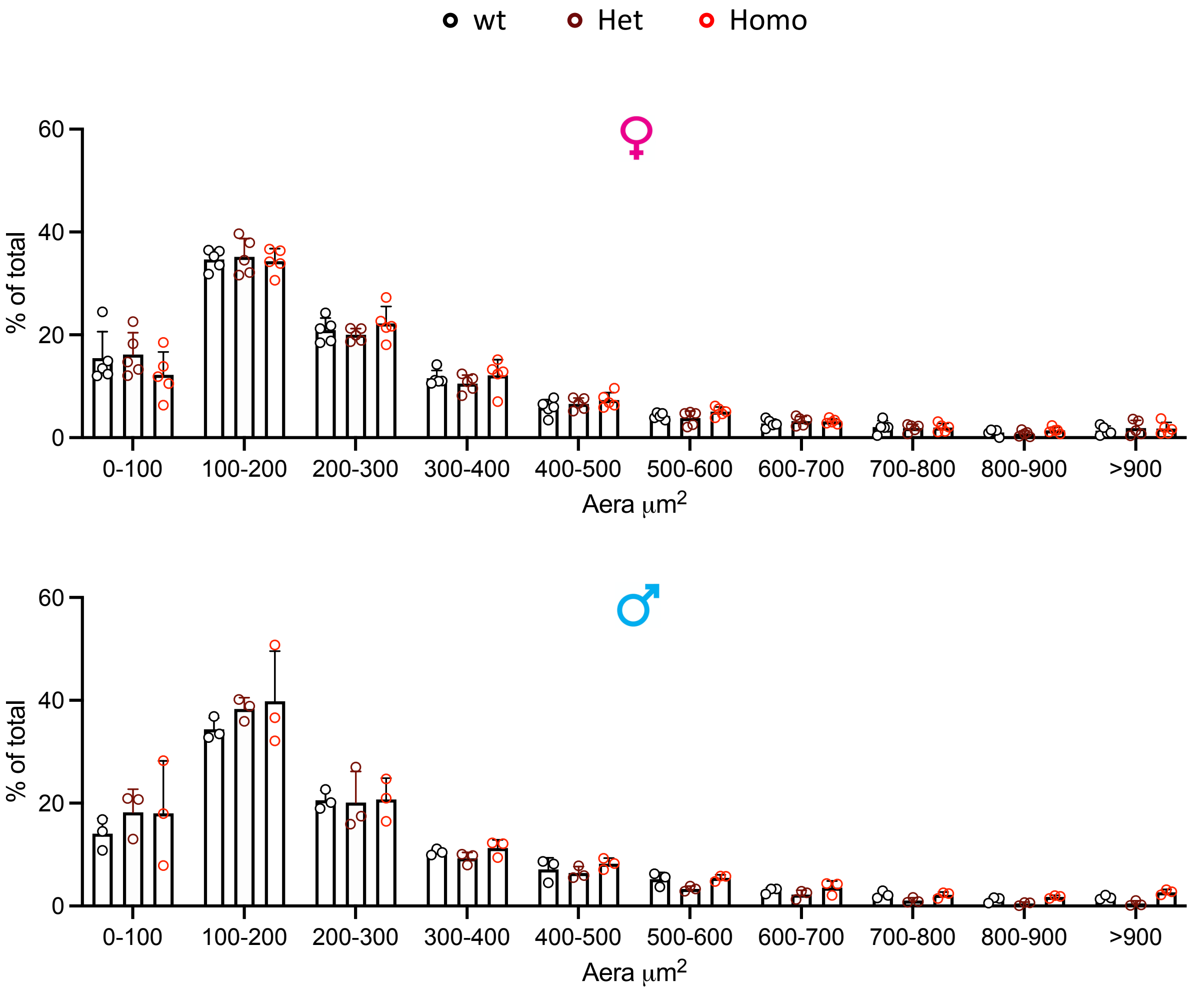

Supplementary Figure 3. Wild-type and mutant mice show a comparable neuron body size distribution in dorsal root ganglia (DRG). DRG neurons were identified by PGP9.5 immunostaining and neuron distribution is represented as the % of neurons in each size category among all neurons. Results are shown as means ± SEM. Females: n=5 mice/genoype; males: n=3 mice/genoype. Two-way ANOVA for genotype and neuron body size. See Supplementary Table 6 for statistics.

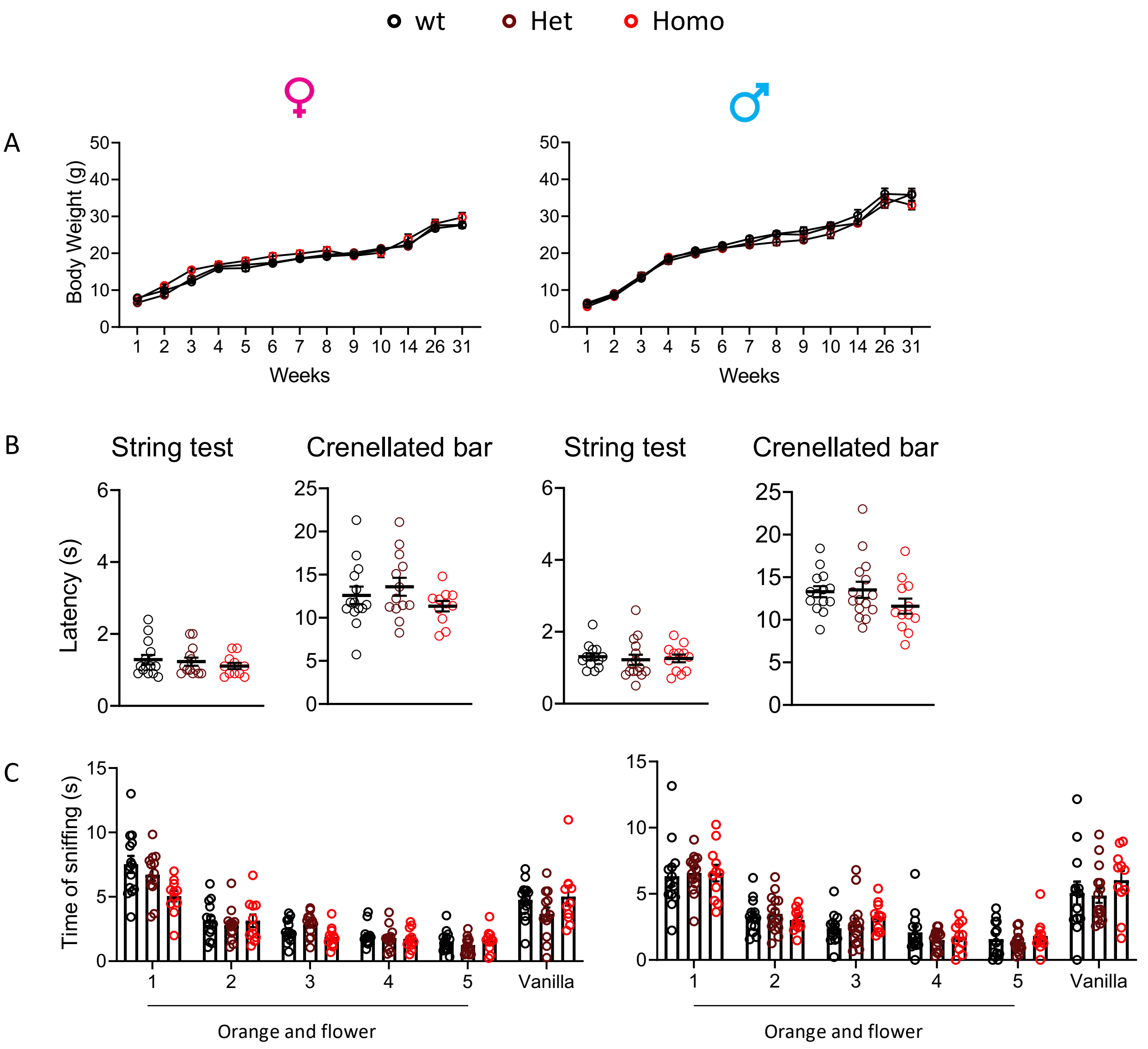

Supplementary Figure 4. *Scn9a*^{R185H} mice show normal healthy condition.

A. Weekly body weight for *Scn9a*^{R185H} females and males indicates no difference between wt and mutant animals. **B.** The string test and the crenellated bar test did not detect any abnormality in muscle strength and motor coordination, respectively. String test, females: wt, n=14; Het, n=13; Homo, n=11; males: wt n=14; Het, n=15; Homo, n=12. Crenellated bar, females: wt n=14; Het, n=13; Homo, n=11; males: wt, n=14; Het, n=15; Homo, n=12. **C.** *Scn9a*^{R185H} mice have normal olfactory discrimination function. Odor discrimination expressed in time spent sniffing odor stimuli in repeated presentations (Trial 1 to 5) of the odor discrimination test. All mice showed change in the time spent sniffing the new olfactory stimuli (vanilla) in the test session. Females: wt, n=13; Het, n=13; Homo, n=11; males: wt n=13; Het, n=15; Homo, n=11. Results are expressed as means ± SEM. Repeated Measures ANOVA and Sidak post-hoc multiple comparison test for body weight and odor discrimination analysis. Het, and Homo were compared to their wt littermates by two tailed Student's t-test or Mann-Whitney test for String and Crenellated bar tests.

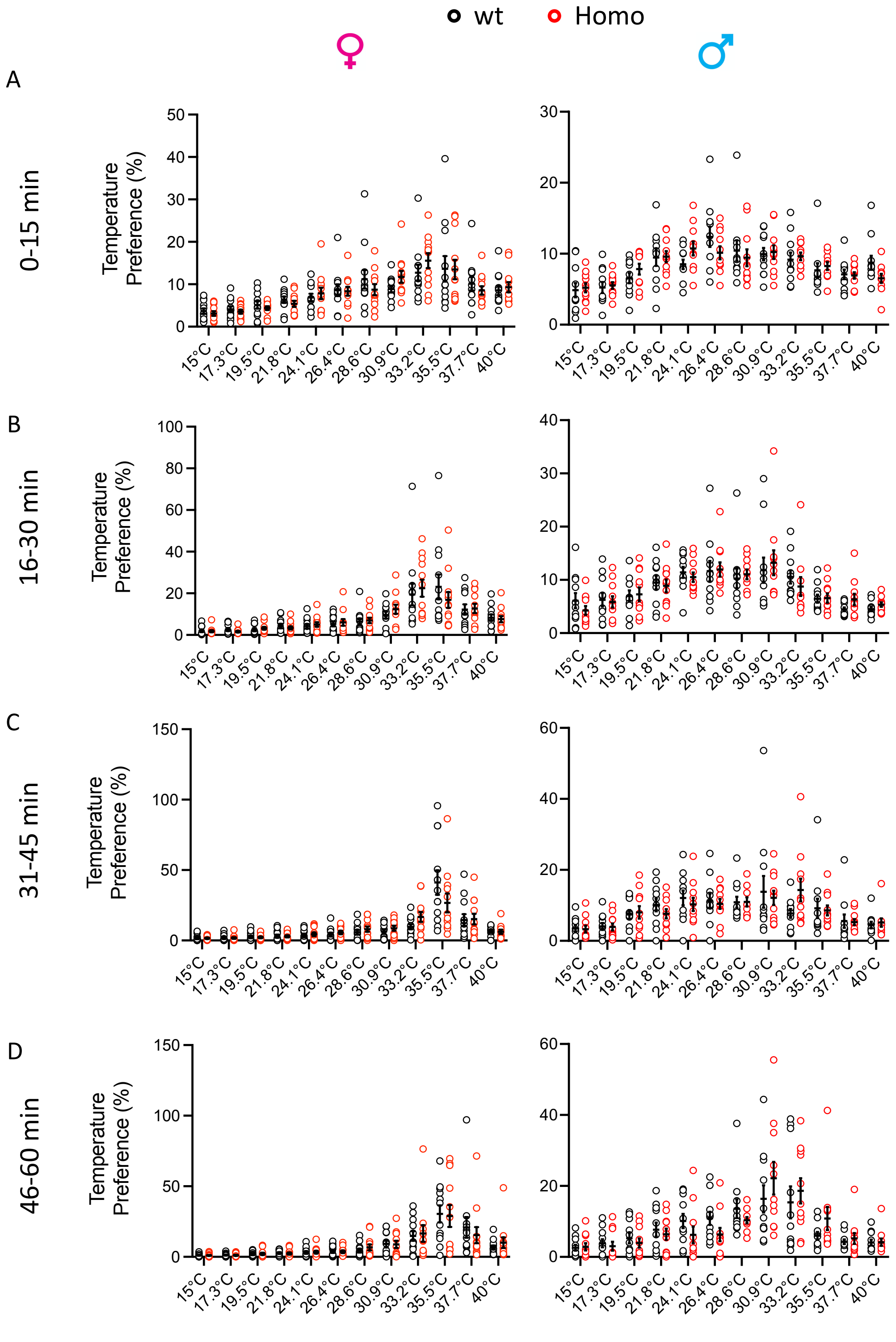

Supplementary Figure 5. ***Scn9a*^{R185H} homozygous mutant mice did not show any difference in temperature preference as compared to *wt* mice in the thermal Thermal Gradient Ring test.** Mice were recorded for 60 min using the 15-40°C environment. Temperature preference was averaged for each 15 min period, **A**. 0-15 min; **B**, 16-30 min; **C**, 31-45 min, **D**, 46-60 min. Temperature preference is shown as the % of time spent above a given temperature. Females: wt, n=12; Homo, n=12; males: wt n=11; Homo, n=11. See Supplementary Table 9 for statistics.

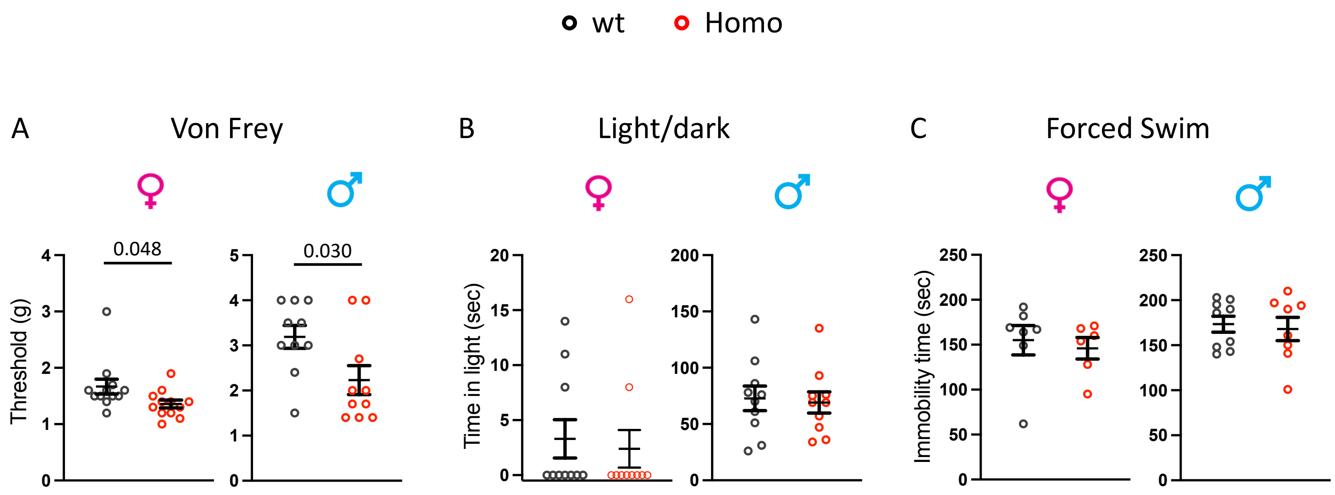

Supplementary Figure 6. Sensitivity to touch and anxio-depressive phenotyping of homozygous *Scn9a*^{R185H} mice tested at INCI institute. A. *Scn9a*^{R185H} mice were more sensitive to touch than wt mice as assessed with the von Frey test. **B-C.** *Scn9a*^{R185H} animals did not show any difference to wt animals when tested for anxiety-like response in the light/dark test and for depression-like behavior in the forced swimming test. Results are shown as means ± SEM. Von Frey: females: wt, n=12; Homo, n=12; males: wt n=10; Homo, n=10. Light/dark: females: wt, n=10; Homo, n=10; males: wt n=10; Homo, n=10. Forced swim: females: wt, n=7; Homo, n=6; males: wt n=9; Homo, n=8. Unpaired two-tailed t-tests. *P* values for genotype differences are shown when significant. See Supplementary Table 11 and 14 for statistics.

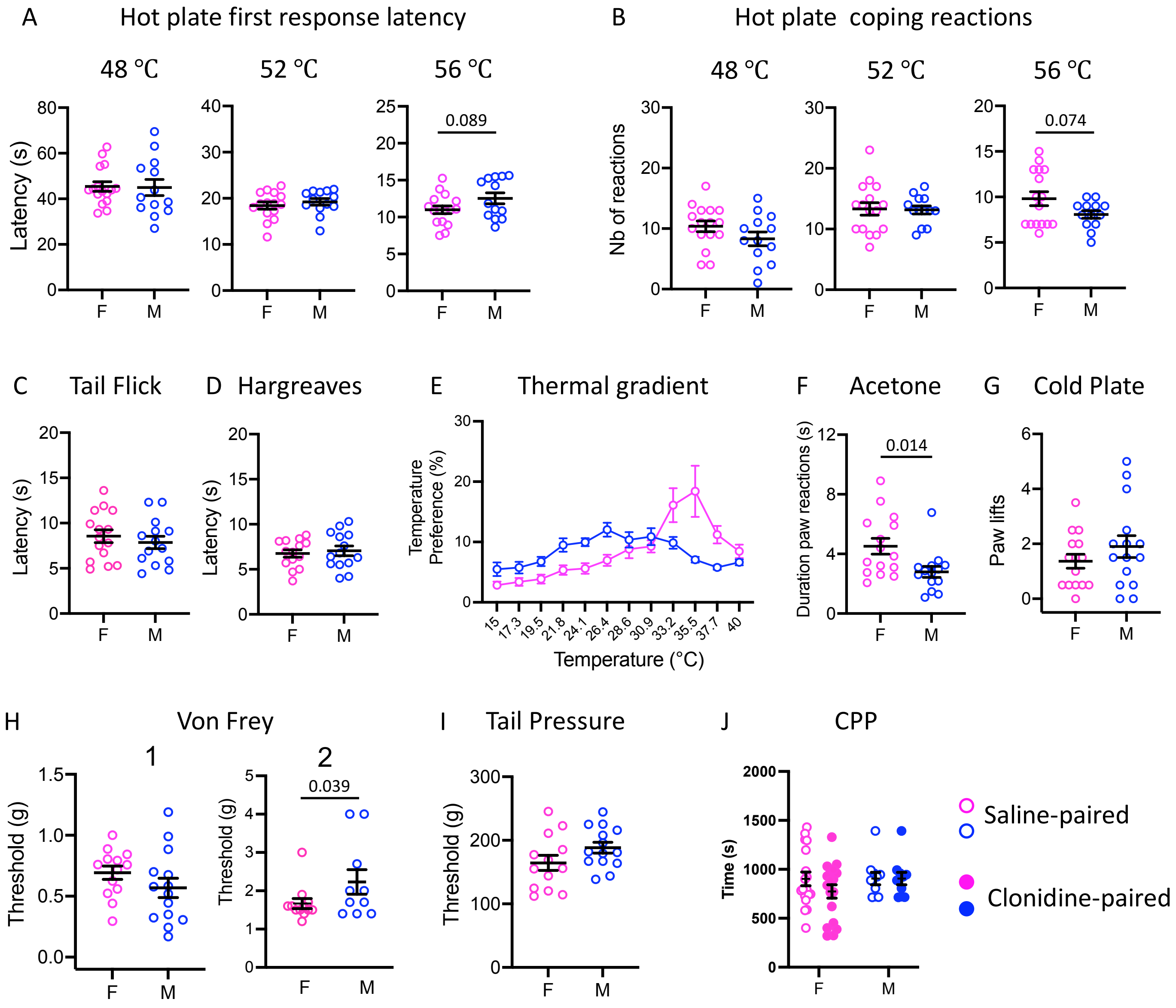

Supplementary figure 7. Influence of sex on pain behaviors in wt mice. A,B) Female wt mice were more sensitive than wt males on the hot plate at 56°C, and **C,D)** No sex difference was found in the tail flick and Hargreaves tests**. E)** Female and male wt mice showed a different temperature profile in the thermal gradient ring**. F)** Wild-type females did more paw reactions to acetone than wt males. G, I) No sex difference was evidenced in the cold plate and tail pressure tests**. H)** In the von Frey model, wt males and females had comparable sensitivity in institute 1 (IGBMC) and wt females were had a lower mechanical threshold than males in institute 2 (INCI). **J)** No sex difference was found in the conditioned place preference (CPP) test for spontaneous pain. Results are shown as means ± SEM. Females: n=12-18/group; males: n=10-13/group. Two-tailed unpaired t-test for hot plate, tail flick, Hargeaves, acetone, cold plate, von Frey and tail pressure tests, two-way ANOVA for the thermal preference ring and CPP. P values for genotype difference are shown when significant or close to significance. See Supplementary Table 13 for statistics.

Supplementary Tables

Supplementary Table 1. Primers used for off-target genomic loci amplification

Gene ID	Forward primer sequence (5´-3´)	Reverse primer sequence (5´-3´)
Exon_Scn3a	crF1 CATTTATTGCTCAAAGACTAAGGGA	crR1AATACTTCACTCACACTAATAAGAA
	crF2 CATACTGTGTATGGCATAGAGTTGA	crR2 GTTGTTGTTAATGCTGTTGTTCTGG
	crF3 AAGTGACTAATGCTGGATTGGCATC	crR3 CTCTAGTTGCTAACTTGGCTTGGGA
	crF4 GAGTTGACCTGATTAACAAGAAAGC	crR4 TCAGCTCTTTAACCTCTTGTCTCTA
Exon_Scn1a	crF1 TGGAGCAGTGGAATGGGTTTACCCT	crR1 GAAACTATGTATGTGATGGATTCTG
	crF2 CGGAGTTCAGGTGCCCAAATCATGC	crR2 CCGAGGACAACATGCTAAGAAGCTG
Exon_Scn11a	crF1TACTCTCAGAGATCCACACAACTTC	crR1 CTCCTTTCTTTCCCAGTCATTTATC
	crF2 CAGAGCCTGGCATGTCAGAGCCACA	crR2 GTTTCCTTAGCCACTTTGCCGTCTC
Exon_Scn4a	crF1 CTGCTAGAGAATGGAAGGACAAGGA	crR1 CATTGTCCCTGTGACACAACACCTT
	crF2 TGGTCTCTGAGAATGCCCTAGATCA	crR2 GGGTAGCCTTATGCTATCTTGGTAC
Exon_Scn5a	crF1 AGTCTATCCTGTCCTCACCTTGCCA	crR1 CGATTGATGACTCTGGTGTGAGGAG
	crF2 GATGAGCCAGGGTGCTCAGACACTT	crR2 CTTCCAGCAAGGGTGAAAGTGGAGA
Intergenic_Tshz3|Zfp536	crF1 GGCTCTGACAATCTTCCTGGTGAAA	crR1 AACCTAAGGAGGCCATTGCATGATG
	crF2 TGCGAAGTTTGGAAACCACCATCCA	crR2 CAGTAGGCTAGACCTCCCACAACAA
Intergenic_Nipsnap3b|Abca1	crF1 CCTCTCCATATCATGTCACTCAGCT	crR1 GTGGCAGAAGGAGAGTGAAGATCCT
	crF2 TCTCCGTGGCACATTTAATCACCGA	crR2 CAGCCATATAGGTGAGCCTGTGCAG

Supplementary Table 2. PCR primers used for on-target genomic loci (*Scn9a*) amplification

Forward primer sequence (5´-3´)	Reverse primer sequence (5´-3´)
crF1 CTTGTGCAATGTACTTTATGAGGTG	crR1 ATCAGGAGACAAGATAATCCTTATG
crF2 ATGCTAACAGCAACCTCCAAGAGAG	crR2 ATAACACATGTCCACTCAGGTACTG
crF3 ATAAACTGTAAAGATTGTAATGAGA	crR3 AATATATGACTGGGAATAGTTAGTC

Supplementary Table 3. Primers and probe used for ddPCR

Genotyping	
Scn9a$^{R185X/wt}$-Forward primer sequence (5´-3´)	AATCCTTGCAAGAGGCTTT
Scn9a$^{R185X/wt}$-Reverse primer sequence (5´-3´)	AAACAATGACAAAGTCCAG
Scn9a^{R185X}-Probe	/56FAM/ACCTTCCTT/ZEN/TGACCCTTGGAACTGG/3IABkFQ/
Scn9a^{wt}-Probe	/56HEX/AATTCACCT/ZEN/TCCTCCGTGACCCTT/3IABkFQ/
**mRNA expression**	
Scn9a^{R185H}-Forward primer sequence (5´-3´)	CATGAGCAACCCTCCAGATT
Scn9a^{R185H}-Reverse primer sequence (5´-3´)	AAACAATGACAACAAAGTCCAG
Scn9a^{R185H}-Probe	/56FAM/ACCTTCCTT/ZEN/TGACCCTTGGAACTGG/3IABkFQ/
Scn9a^{wt}-Probe	/56HEX/AATTCACCT/ZEN/TCCTCCGTGACCCTT/3IABkFQ/
Scn9a$^{R185X/wt}$-Forward primer sequence (5´-3´)	CATGAGCAACCCTCCAGATT
Scn9a$^{R185X/wt}$-Reverse primer sequence (5´-3´)	AAACAATGACAACAAAGTCCAG
Scn9a$^{R185X/wt}$-Probe	56-FAM/ACCTTCCTT/ZEN/TGACCCTTGGAACTGG/3IABkFQ/
Scn9a^{wt}-Probe	/56HEX/AATTCACCT/ZEN/TCCTCCGTGACCCTT/3IABkFQ/
Hprt-Forward primer sequence (5´-3´)	CCCCAAAATGGTTAAGGTTGC
Hprt -Reverse primer sequence (5´-3´)	AACAAAGTCTGGCCTGTATCC
Hprt -Probe	5HEX/CTTGCTGGT/ZEN/GAAAAGGACCTCTCGAA/3IABkFQ/

Supplementary Table 4. Potential off-target analysis sequence

Gene ID	Potential off-target sequences	Mismatches	locus
Scn9a (on-target gene)	GCCAGTTCCAAGGGTCACGG AGG	0	chr2:66563568-66627708
Exon_Scn3a	GCCAGTTCCATGGGTCACGA AGA	2	chr2:65526522-65526544
Exon_Scn1a	GCCAGTTCCATGGGTCGCGA AGG	3	chr2:66334233-66334255
Exon_Scn11a	GCCAGTTCCAAGGATCTCGG AGG	2	chr9:119813083-119813105
Exon_Scn4a	GCCAGTTCCAGGGGTCTCGG AGG	2	chr11:106348297-106348319
Exon_Scn5a	GCCAGTTCCACGGGTCCCGG AGG	2	chr9:119550631-119550653
Intergenic_Tshz3|Zfp536	GGCAGATCCAAGGGTCACGG AGA	2	chr7:36801277-36801299
Intergenic_Nipsnap3b|Abca1	CCCAGTTCCAAGGGTCACGC TGA	2	chr4:53023676-53023698

Supplementary Table 5. Expression of *Scn9a* transcripts in wt and *Scn9a*^{R185H} mutant mice

Figure	Test	Analysis	Groups	Statistics
Fig. 1C	DRG wt allele	Two-way ANOVA	Genotype	**P<0.001** F (2, 18) = 689.9
			Sex	P=0.819 F (1, 18) = 0.05364
			Interaction	P=0.580 F (2, 18) = 0.5615
	Spinal cord wt allele	Two-way ANOVA	Genotype	**P<0.001** F (2, 24) = 66.82
			Sex	P=0.647 F (1, 24) = 0.2148
			Interaction	P=0.926 F (2, 24) = 0.07724
Fig. 1C	DRG mutant allele	Two-way ANOVA	Genotype	**P<0.001** F (2, 18) = 1321
			Sex	P=0.262 F (1, 18) = 1.339
			Interaction	P=0.498 F (2, 18) = 0.7251
	Spinal cord mutant allele	Two-way ANOVA	Genotype	**P<0.001** F (2, 24) = 94.35
			Sex	P=0.296 F (1, 24) = 1.141
			Interaction	P=0.532 F (2, 24) = 0.647

Supplementary Table 6. Nav1.7 protein expression in DRG and sciatic nerves

Figure	Test	Analysis	Groups	Statistics
Fig. 2B	Nav1.7-positive neurons in female DRGs	Two-way ANOVA	Genotype	P=0.715<br>F (2, 120) = 0.3358
			Size	**P<0.001**<br>F (9, 120) = 267.9
			Interaction	P=0.416<br>F (18, 120) = 1.045
	Nav1.7-positive neurons in male DRGs	Two-way ANOVA	Genotype	P=0.787<br>F (2, 60) = 0.2402
			Size	**P<0.001**<br>F (9, 60) = 135.4
			Interaction	P=0.934<br>F (18, 60) = 0.5260
	Total neurons in female DRGs	Two-way ANOVA	Genotype	P=0.905<br>F (2, 120) = 0.09932
			Size	**P<0.001**<br>F (9, 120) = 382.9
			Interaction	P=0.560<br>F (18, 120) = 0.9165
	Total neurons in male DRGs	Two-way ANOVA	Genotype	P=0.172<br>F (2, 60) = 1.815
			Size	**P<0.001**<br>F (9, 60) = 116.0
			Interaction	P=0.980<br>F (18, 60) = 0.4126
Fig. 2D	Sciatic nerves	Two-way ANOVA	Genotype	P=0.384<br>F (2, 17) = 1.014
			Sex	**P=0.009**<br>F (1, 17) = 8.692
			Interaction	P=0.724<br>F (2, 17) = 0.328
		Females One-way ANOVA	Genotype	P=0.445<br>F(2, 9) = 0.8858
		Males One-way ANOVA	Genotype	P=0.850<br>F(2, 8) = 0.1654

Supplementary Table 7. Responses of wt and *Scn9a*^{R185H} mutant mice in the hot plate test

Figure	Test	Analysis	Groups	Statistics
Fig. 3A	First response latency 48°C	Two-way ANOVA	Genotype	P=0.729<br>F (2, 79) = 0.3178
			Sex	P=0.890<br>F (1, 79) = 0.01915
			Interaction	P=0.450<br>F (2, 79) = 1.8076
	First response latency 52°C	Two-way ANOVA	Genotype	P=0.949<br>F (2, 78) = 0.05248
			Sex	P=0.931<br>F (1, 78) = 0.0076
			Interaction	P=0.667<br>F (2, 78) = 0.4076
	First response latency 56°C	Two-way ANOVA	Genotype	P=0.211<br>F (2, 79) = 1.586
			Sex	P=0.061<br>F (1, 79) = 3.625
			Interaction	P=0.777<br>F (2, 79) = 0.2537
Fig. 3B	Jump latency 48°C	Two-way ANOVA	Genotype	P=0.224<br>F (2, 79) = 1.523
			Sex	P=0.090<br>F (1, 79) = 2.938
			Interaction	P=0.224<br>F (2, 79) = 1.523
	Jump latency 52°C	Two-way ANOVA	Genotype	P=0.339<br>F (2, 79) = 1.096
			Sex	**P=0.0096**<br>F (1, 79) = 7.045
			Interaction	P=0.384<br>F (2, 79) = 0.9681
		Females<br>One-way ANOVA	Genotype	P=0.306<br>F (2, 44) = 1.218
		Males<br>One-way ANOVA	Genotype	P=0.174<br>F (2, 35) = 1.837
	Jump latency 56°C	Two-way ANOVA	Genotype	P=0.077<br>F (2, 79) = 2.642
			Sex	**P=0.045**<br>F (1, 79) = 4.145
			Interaction	**P=0.040**<br>F (2, 79) = 3.347
		Females<br>One-way ANOVA	Genotype	**P=0.008**<br>F (2, 44) = 5.459
		Dunnett's multiple comparison test	wt vs homo	**P=0.004**
			wt vs het	P=0.365
		Males<br>One-way ANOVA	Genotype	P=0.479<br>F (2, 35) = 0.751

Supplementary Table 7 (continued). Responses of wt and *Scn9a*^{R185H} mutant mice in the hot plate test

Figure	Test	Analysis	Groups	Statistics
Fig. 3C	Coping reactions 48°C	Two-way ANOVA	Genotype<br>Sex<br>Interaction	p=0.268<br>F (2, 79) = 1.340<br>P=0.431<br>F (1, 79) = 0.6261<br>P=0.350<br>F (2, 79) = 1.064
	Coping reactions 52°C	Two-way ANOVA	Genotype<br>Sex<br>Interaction	P=0.061<br>F (2, 79) = 2.896<br>P=0.162<br>F (1, 79) = 1.993<br>P=0.138<br>F (2, 79) = 2.031
		Females<br>One-way ANOVA	Genotype	**P=0.032**<br>F (2, 44) = 3.739
		Dunnett's multiple comparison test	wt vs homo<br>wt vs het	**P=0.021**<br>P=0.687
		Males<br>One-way ANOVA	Genotype	P=0.842<br>F (2, 35) = 0.1722
	Coping reactions 56°C	Two-way ANOVA	Genotype<br>Sex<br>Interaction	**P=0.020**<br>F (2, 79) = 4.091<br>**P<0.001**<br>F (1, 79) = 11.79<br>P=0.972<br>F (2, 79) = 0.0287
		Females<br>One-way ANOVA	Genotype	P=0.169<br>F (2, 44) = 1.853
		Males<br>One-way ANOVA	Genotype	P=0.074<br>F (2, 35) = 2.805
		Dunnett's multiple comparison test	wt vs homo<br>wt vs het	P=0.077<br>P=0.995

Supplementary Table 8. Sensitivity to *Scn9a*^{R185H} mutant mice in the tail flick and Hargreaves tests

Figure	Test	Analysis	Groups	Statistics
Fig. 3D	Tail Flick	Two-way ANOVA	Genotype	P=0.147<br>F (2, 77) = 1.966
			Sex	P=0.350<br>F (1, 77) = 0.8837
			Interaction	P=0.279<br>F (2, 77) = 1.298
		Females<br>One-way ANOVA	Genotype	**P=0.050**<br>F (2, 41) = 3.226
		Dunnett's multiple comparison test	wt vs het<br>wt vs homo	P=0.102<br>**P=0.042**
		Males<br>One-way ANOVA	Genotype	P=0.803<br>F (2, 36) = 0.220
Fig. 3E	Hargreaves	Two-way ANOVA	Genotype	P=0.294<br>F (2, 72) = 1.246
			Sex	P=0.157<br>F (1, 72) = 2.044
			Interaction	P=0.882<br>F (2, 72) = 0.1253
		Females<br>One-way ANOVA	Genotype	P=0.637<br>F (2, 35) = 0.456
		Males<br>One-way ANOVA	wt vs het	P=0.476<br>F (2, 37) = 0.7579

Supplementary Table 9. Thermal preference in *Scn9a*^{R185H} mutants and wt animals

Figure	Test	Analysis	Groups	Statistics
Fig. 3F	Females 30 min	Two-way ANOVA	Genotype	P=0.998<br>F (1, 264) = 4.63e-006
			Temperature	**P<0.001**<br>F (11, 264) = 19.60
			Interaction	P=0.865<br>F (11, 264) = 0.5538
Fig. 3F	Males 30 min	Two-way ANOVA	Genotype	P=0.995<br>F (1, 240) = 3.96e-005
			Temperature	**P<0.001**<br>F (11, 240) = 13.44
			Interaction	P=0.985<br>F (11, 240) = 0.2997
Suppl. Fig. 4A	Males 0-15 min	Repeated measures Two-way ANOVA	Genotype x temperature zone	P=0.494<br>F (11, 220) = 0.9496
Suppl. Fig. 4B	Males 16-30 min	Repeated measures Two-way ANOVA	Genotype x temperature zone	P=0.969<br>F (11, 220) = 0.3628
Suppl. Fig. 4C	Males 31-45 min	Repeated measures Two-way ANOVA	Genotype x temperature zone	P=0.740<br>F (11, 220) = 0.6976
Suppl. Fig. 4D	Males 46-60 min	Repeated measures Two-way ANOVA	Genotype x temperature zone	P=0.475<br>F (11, 220) = 0.969
Suppl. Fig. 4A	Females 0-15 min	Repeated measures Two-way ANOVA	Genotype x temperature zone	P=0.804<br>F (11, 242) = 0.6281
Suppl. Fig. 4B	Females 16-30 min	Repeated measures Two-way ANOVA	Genotype x temperature zone	P=0.925<br>F (11, 242) = 0.4625
Suppl. Fig. 4C	Females 31-45 min	Repeated measures Two-way ANOVA	Genotype x temperature zone	P=0.167<br>F (11, 242) = 1.4145
Suppl. Fig. 4D	Females 46-60 min	Repeated measures Two-way ANOVA	Genotype x temperature zone	P=0.998<br>F (11, 242) = 0.1947

Supplementary Table 10a. Sensitivity to acetone in wt and *Scn9a*^{R185H} mutant mice

Figure	Test	Analysis	Groups	Statistics
Fig. 4A-B Acetone test	Number of paw reactions	Two-way ANOVA	Genotype	**P=0.024** F (2, 74) = 3.907
			Sex	**P<0.001** F (1, 74) = 25.32
			Interaction	**P=0.048** F (2, 74) = 3.17
		Females One-way ANOVA	Genotype	P=0.605 F (2, 38) = 0.5098
		Males One-way ANOVA	Genotype	**P=0.002** F (2, 36) = 7.357
		Males Dunnett's multiple comparison test	wt vs het	P=0.189
			wt vs homo	P=0.054
	Duration of paw reactions	Two-way ANOVA	Genotype	**P=0.027** F (2, 74) = 3.794
			Sex	**P<0.001** F (1, 74) = 16.09
			Interaction	P=0.118 F (2, 74) = 2.204
		Females One-way ANOVA	Genotype	P=0.273 F (2, 38) = 0.1342
		Males One-way ANOVA	Genotype	**P=0.004** F (2, 36) = 6.548
		Males Dunnett's multiple comparison test	wt vs het	P=0.838
			wt vs homo	**P=0.011**

Supplementary Table 10b. Sensitivity to cold plate of wt and *Scn9a*^{R185H} mutant mice

Figure	Test	Analysis	Groups	Statistics
Fig. 4C	Number of paw lifts	Two-way ANOVA	Genotype	P=0.082 F (2, 78) = 2.583
			Sex	P=0.712 F (1, 78) = 0.1373
			Interaction	P=0.509 F (2, 78) = 0.6816
		Females One-way ANOVA	Genotype	P=0.528 F (2, 40) = 0.6497
		Males One-way ANOVA	Genotype	P=0.125 F (2, 38) = 2.200
		Dunnett's multiple comparison test	wt vs het<br>wt vs homo	P=0.813<br>P=0.087

Supplementary Table 11. Sensitivity to mechanical stimuli in wt and *Scn9a*^{R185H} mice

Figure	Test	Analysis	Groups	Statistics
Fig. 4D	Von Frey IGBMC	Two-way ANOVA	Genotype	**P=0.0012**<br>F (2, 68) = 7.413
			Sex	P=0.196<br>F (1, 68) = 1.707
			Interaction	P=0.719<br>F (2, 68) = 0.331
		Females<br>One-way ANOVA	Genotype	**P=0.015**<br>F (2, 32) = 4.769
		Males<br>One-way ANOVA	Genotype	P=0.053<br>F (2, 36) = 1.520
		Females<br>Dunnett's multiple comparison test	wt vs het	**P=0.039**
			wt vs homo	**P=0.016**
		Males<br>Dunnett's multiple comparison test	wt vs het	P=0.365
			wt vs homo	**P=0.030**
Suppl Fig. 6A	Von Frey INCI	Two-way ANOVA	Genotype	**P=0.003**<br>F (1, 40) = 9.834
			Sex	**P<0.001**<br>F (1, 40) = 35.06
			Interaction	P=0.115<br>F (1, 40) = 2.596
		Sidak's multiple comparison test	Females wt vs homo	P=0.460
			Males wt vs homo	**P=0.005**
		Two-tailed unpaired t-test	Females wt vs homo	**P=0.048**
			Males wt vs homo	**P=0.030**
Fig. 4E	Tail Pressure	Two-way ANOVA	Genotype	P=0.501<br>F (2, 70) = 0.6975
			Sex	P=0.343<br>F (1, 70) = 0.9100
			Interaction	P=0.181<br>F (2, 70) = 0.1.752

Supplementary Table 12. Spontaneous pain in *Scn9a*^{R185H} mice as assessed in the CCP paradigm

Fig. 6A	Females	Three-way repeated measures ANOVA	Genotype x solution-paired (Saline vs Clonidine) x trial (Pre-test vs Test)	**P=0.026**<br>F (1, 68) = 5.149
Fig. 6B	Males	Three-way repeated measures ANOVA	Genotype x solution-paired (Saline vs Clonidine) x trial (Pre-test vs Test)	**P=0.002**<br>F (1, 36) = 10.768
Fig. 6A	Wt Females	Two-way repeated measures ANOVA	Treatment	P=0.213<br>F (1, 34) = 1.610
			Trial	P=0.597<br>F (1, 34) = 0.3298
			Interaction	P=0.453<br>F (1, 34) = 0.6237
Fig. 6A	Homo Females	Two-way repeated measures ANOVA	Treatment	**P<0.001**<br>F (1, 34) = 14.82
			Trial	P=0.727<br>F (1, 34) = 0.1239
			Interaction	**P<0.001**<br>F (1, 34) = 16.18
Fig. 6B	Wt Males	Two-way repeated measures ANOVA	Treatment	P=0.388<br>F (1, 18) = 0.7823
			Trial	P=0.377<br>F (1, 18) = 0.8219
			Interaction	P=0.667<br>F (1, 18) = 0.1914
Fig. 6B	Homo Males	Two-way repeated measures ANOVA	Treatment	**P=0.006**<br>F (1, 18) = 9.480
			Trial	P=0.571<br>F (1, 18) = 0.8219
			Interaction	**P<0.001**<br>F (1, 18) = 23.17

Supplementary Table 13. Influence of sex on pain behaviors in wt mice

Figure	Test	Analysis	Test	Statistics
Suppl. Fig.7A	Hot plate First response latency	48°C<br>52°C<br>56°C	t-test<br>t-test<br>Mann Whitney	P=0.910<br>P=0.463<br>P=0.166
	Hot plate Jump latency	52°C<br>56°C	Mann Whitney<br>Mann-whitney	P=0.488<br>P=0.652
Suppl. Fig.7B	Hot plate Coping reactions	48°C<br>52°C<br>56°C	t-test<br>t-test<br>t-test	P=0.156<br>P=0.903<br>P=0.074
Suppl. Fig.7C	Tail flick		t-test	P=0.485
Suppl. Fig.7D	Hargreaves		t-test	P=0.663
Suppl. Fig.7E	Thermal gradient	Two-way ANOVA	Sex<br>Temperature<br>Interaction	P=0.981<br>F (1, 252) = 0.00056<br>P<0.001<br>F (1, 252) = 7.917<br>P<0.001<br>F (1, 252) = 6.390
Suppl. Fig.7F	Acetone		t-test	**P=0.014**
Suppl. Fig.7G	Cold plate		t-test	P=0.271
Suppl. Fig.7H	Von Frey IGBMC		t-test	P=0.215
Suppl. Fig.7H	Von Frey INCI		t-test	**P=0.039**
Suppl. Fig.7I	Tail pressure		t-test	P=0.110
Suppl. Fig.JF	CPP Clonidine vs saline test	Two-way ANOVA	Sex<br>Treatment<br>Interaction	P=0.368<br>F (1, 52) = 0.825<br>P=0.394<br>F (1, 52) = 0.7402<br>P=0.394<br>F (1, 52) = 0.7402

Supplementary Table 14. Anxio-depressive behaviors in *Scn9a*^{R185H} mice.

Figure	Test	Analysis	Groups	Statistics
Suppl. Fig. 6B	Light/dark	Females, wo tailed unpaired t-test	genotype	P=0.716
Suppl. Fig. 4B	Light/dark	Males, wo tailed unpaired t-test	genotype	P=0.801
Suppl. Fig. 6C	Forced swim	Females, wo tailed unpaired t-test	genotype	P=0.675
Suppl. Fig. 6C	Forced swim	Males, wo tailed unpaired t-test	genotype	P=0.733
